# Towards the design of artificial sensing materials via quantum-informed explainable AI

**DOI:** 10.1186/s13321-026-01232-3

**Published:** 2026-05-29

**Authors:** Li Chen, Leonardo Medrano Sandonas, Shirong Huang, Alexander Croy, Gianaurelio Cuniberti

**Affiliations:** 1 Institute for Materials Science and Max Bergmann Center for Biomaterials, TUD Dresden University of Technology, 01062 Dresden, Germany; 2 Institute of Physical Chemistry, Friedrich Schiller University Jena, 07737 Jena, Germany; 3 Dresden Center for Computational Materials Science (DCMS), TUD Dresden University of Technology, 01062 Dresden, Germany; 4 Cluster of Excellence CARE, TUD Dresden University of Technology and RWTH Aachen, Dresden, Germany; 5 Cluster of Excellence CeTI, TUD Dresden University of Technology, Dresden, Germany

**Keywords:** Quantum-informed descriptors, Molecular sensing, Explainable AI, Healthcare

## Abstract

Computational design of sensing materials remains fundamentally challenging due to the vast configurational landscape and the absence of robust property correlations that enable efficient molecular representation. These challenges become particularly critical in healthcare applications, where reliable and interpretable molecular recognition is essential for non-invasive diagnostics. To address these challenges, we developed MORE-ML, a quantum-informed AI framework that combines electronic-structure-derived properties of e-nose molecular building blocks with machine learning (ML) methods to uncover sensing mechanisms and guide the design of new systems. Within this framework, we expanded our previous dataset, MORE-Q, to MORE-QX by sampling a larger conformational space of interactions between body odor volatilomes (BOV) molecules and mucin-derived receptors, both in the gas phase and when deposited on graphene. MORE-QX provides extensive electronic binding features (BFs) computed upon BOV adsorption. Analysis of the property space revealed weak correlations between quantum-mechanical (QM) properties of building blocks and resulting BFs. Leveraging this observation, we defined electronic descriptors of building blocks as inputs for tree-based ML models to predict BFs. Benchmarking showed CatBoost models outperform alternatives, especially in transferability to unseen compounds. Through explainable AI, we reduced the high-dimensional QM property space to a compact and physically interpretable set of descriptors, revealing the properties that most influence BF predictions. Collectively, MORE-ML combines QM insights with ML to provide mechanistic understanding and rational design principles for artificial sensing materials in BOV sensing. This approach establishes a foundation for advancing materials capable of analyzing complex odor mixtures, bridging the gap between molecular-level computations and practical e-nose applications.

**Supplementary Information:**

The online version contains supplementary material available at 10.1186/s13321-026-01232-3.

## Scientific contribution

We developed the MORE-ML framework, which integrates electronic-structure-derived molecular descriptors with interpretable tree-based modeling and SHAP analysis to uncover physically and chemically grounded design principles for artificial sensing materials. In this context, we introduced the MORE-QX dataset, an extensive collection of electronic binding features characterizing the selectivity and sensitivity of a broad range of odorant–receptor conformations. Our work establishes an AI-assisted strategy for rational receptor engineering and identifies the key quantum-mechanical features governing binding behavior, thereby providing a more transparent and navigable pathway through the largely unexplored receptor design space.

**Supplementary Information:**

The online version contains supplementary material available at 10.1186/s13321-026-01232-3.

## Introduction

The rapid advancement in artificial intelligence (AI) has significantly accelerated the development of AI-driven technologies, enabling precise recognition of objects, faces, voices, and tactile sensations [1, 2]. Despite these advancements, a considerable technological gap persists in effectively interpreting and predicting the chemical environment surrounding humans. To bridge this gap, customized electronic noses have emerged, demonstrating notable proficiency in detecting volatile organic compounds (VOCs) [3–6]. Specifically, VOCs emitted from the human body (referred to as body odor volatilome (BOV)) act as unique chemical fingerprints and hold great promise for healthcare applications [7], e.g., serving as biomarkers for Alzheimer’s and Parkinson’s diseases [8, 9]. However, there remains a strong and persistent need for rapid and reliable sensing materials capable of detecting biomarkers [10] e.g., BOV molecules within digital olfactory systems, particularly for medical diagnostics.

Inspired by the sensitivity [11] and the discriminative power of the human olfactory system [12], diverse molecular olfactory receptors have recently been synthesized (e.g., mucin-derived receptors [13–15]). This progress has driven the development of experimental protocols aimed at controlling receptor affinity toward BOV molecules in gas sensing by incorporating specific functional groups with varying chemical characteristics on glaco-conjugated [16]. However, obtaining detailed information on BOV–receptor interactions—and thus guidance for receptor optimization—remains both costly and time-consuming when relying on empirical trial-and-error screening. This indicates that a key limitation of current prototype receptors lies in the lack of mechanistic insight into their sensitivity and selectivity across the vast chemical space of BOV–receptor systems. This bottleneck underscores the need for sustainable strategies to rationally design high-performance receptor-based biomimetic sensors. Similar to the transformative impact of molecular electronics a few decades ago [17], quantum-mechanical (QM) methodologies could revolutionize the field of chemical sensing by providing a deeper understanding of the physical and chemical interactions that govern key performance metrics such as recovery time, charge transfer, and Schottky barrier potential [18]. Furthermore, integrating QM-derived property data with AI techniques has the potential to yield reliable and efficient computational frameworks for guiding the design of materials for sensors with high sensitivity and selectivity—an approach that has recently proven successful in drug discovery studies [19–22].

Within this context, we have recently introduced the MORE-Q dataset [23], providing, for the first time, an extensive set of QM property data corresponding to the atomistic building blocks of artificial olfactory molecular sensors: BOV molecules, mucin-derived olfactorial receptors, and BOV-receptor dimer systems. MORE-Q also contains electronic structure data describing the intermolecular interactions between the most stable dimer systems and a graphene surface. All together, this dataset enables the exploration of key binding features (BFs) induced by BOV adsorption such as adsorption energy [24, 25], charge transfer [26], and work function change [27]. This collection of BFs represent a big step towards the rational design and optimization of BOV–receptor systems due to the comprehensive electronic description of sensing performance; however, there are still some additional challenges to address before developing a sustainable framework for BOV–receptor design. For instance, analogous to ligand-pocket motifs [28, 29], the sensing process is inherently dynamic and governed by weak non-covalent interactions (electrostatics and hydrogen bonding), indicating a structural flexibility that yield a rugged energy landscape with myriad local minima and versatile binding configurations [30]. On the other hand, current theoretical models lack the quantitative rigor required to quantitatively delineate property–property and structure–property relationships, hindering a clear understanding of the role of sensing building blocks in BF behavior.

A promising approach to elucidate the complex mappings between atomic structures and BFs is the sustainable use of machine learning (ML) methods [31]. For instance, Ulissi et al. recently introduced the AdsorbML framework [32], which integrates heuristic search with ML potentials to accelerate gas–metal adsorption energy calculations, achieving both high predictive accuracy and substantial computational speedups compared to conventional density functional theory (DFT). Similarly, GAME-Net [33], a graph neural network model, was developed to predict adsorption energies of organic molecules on catalytic surfaces with near-DFT accuracy, reaching errors of 0.18 eV (0.016 eV per atom) for large biomass and plastic fragments. More recently, Chen et al. introduced AdsMT [34], a multimodal Transformer that combines catalyst surface graph representations with adsorbate feature vectors through a cross-attention mechanism to predict global minimum adsorption energies without enumerating adsorption sites. While various ML-based studies [35–39] have focused on the adsorption of small and simple adsorbates (e.g., ${\hbox {O}_2}$, ${\hbox {CO}_2}$, and ${\hbox {H}_2}$) on flat metal surfaces, other electronic BFs, such as charge transfer and work function change, have been less explored. In addition, for large interacting molecules like the BOV-receptor systems, the concepts of binding site and adsorption distance become ill-defined owing to complex interaction morphologies and configurational polymorphism, which makes the development of predictive models more challenging. Furthermore, most of these works prioritize achieving high predictive accuracy, often at the expense of model interpretability, thereby limiting the physical and chemical insights that can be derived from these complex mappings. This lack of explainability also affects the exploration of the binding features space, where the optimization of one feature offers no guarantee of concurrent improvements in others, complicating further the rational design of sensing materials.

**Fig. 1:**
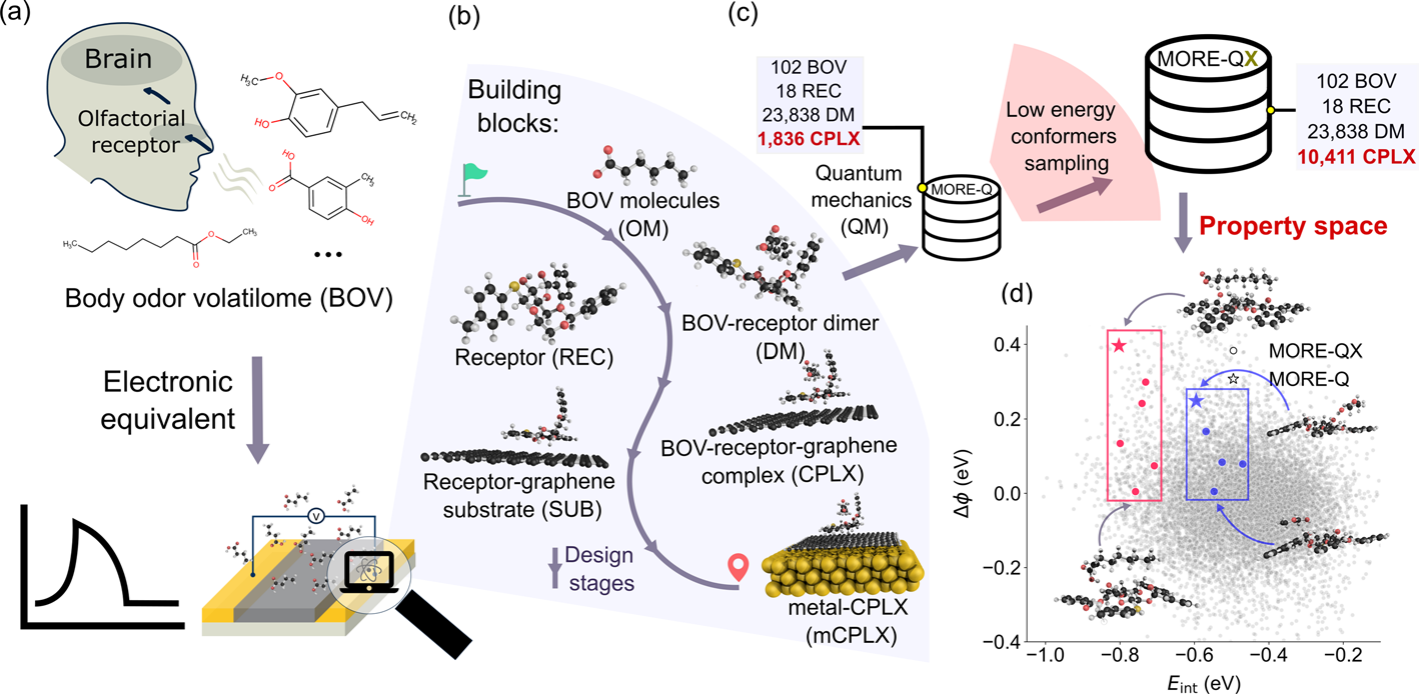
The schematic workflow for **M**olecular **O**lfactorial **R**eceptor **E**ngineering by **Q**uantum mechanics (MORE-Q) [23] dataset expansion to MORE-QX dataset. **a** The bio-electronic noses (top right panel) are designed as an electronic equivalent to the olfactory system (top left panel), e.g., for sensing BOV molecules (or odorant molecules, OM). **b** The building blocks at different design stages for the bio-mimetic sensor from QM perspective including OM molecules, molecular receptor (REC), OM-REC dimer molecule (DM), REC-graphene substrate (SUB), OM-REC-graphene complex system (CPLX) and eventually these systems deposited on the gold electrode (mCPLX). These abbreviations are used throughout this manuscript. **c** The QM properties of the relevant building blocks were calculated and incorporated into the MORE-Q dataset, which includes monomer systems of 102 OM and 18 REC molecules, 23,838 DM systems, and 1,836 CPLX systems derived from the most stable DM configurations. Sampling multiple low-energy DM conformers expanded the CPLX subset, yielding the MORE-QX dataset with 10,411 CPLX systems. **d** 2D projection of the high-dimensional MORE-QX property space defined by the work function change $\Delta \phi$ and DM interaction energy $E_\mathrm{int}$. The conformers of two DM systems (red and blue) are labeled, where the most stable one (MORE-Q) is marked as star while the other low-energy conformers (MORE-QX) are marked with circles. The atomic structure associated to the maximal and minimal values are depicted on the plot for each DM system

To address these challenges, we develop the MORE-ML framework, which integrates QM-derived molecular properties with ML methods to investigate how structural, global, and atomic-level features of electronic-nose building blocks influence BOV adsorption. By doing so, we seek to clarify the sensing mechanism and formulate design principles for BOV–receptor systems in artificial sensing materials. To approximate the thermodynamic ensemble, we expanded the MORE-Q dataset [23] into MORE-QX by sampling multiple low-energy BOV–receptor dimer (DM) conformers adsorbed on graphene. This process increased the number of BOV–receptor–graphene complexes from 1,836 to 10,441 (see Fig. 1). A comprehensive analysis of MORE-QX reveals that DM conformers with similar binding energies can nevertheless show markedly different BFs such as charge transfer and work function change. Furthermore, DM properties and BFs exhibit only weak to moderate correlations, even though these properties were chosen following fundamental physical and chemical principles. Despite particularly weak correlations among BFs, we retain flexibility in identifying systems that share a similar set of electronic binding characteristics—clear evidence for the existence of “Freedom of design” in the MORE-QX property space [40]. To enable rapid and accurate navigation of the binding feature space–and thereby support practical design of BOV–receptor complexes for sensing–we develop tree-based regression models that map QM-derived property data of building blocks to their associated BFs. Within MORE-ML, we further exploit the interpretability of these models using SHapley Additive Explanations (SHAP) [41] to extract mechanistic insights into the sensing process. Overall, this work provides quantum-informed understanding of adsorption mechanisms and BOV–receptor-graphene interactions, paving the way for controlled and robust discovery of artificial sensing materials.

## Computational methods

### MORE-QX dataset generation

The intrinsic flexibility of large molecular receptors (REC) brings a crucial factor to consider in the understanding of the sensing mechanism in artificial olfactory sensors. Analogous to the binding process of ligands into protein pockets [28, 29], the interaction between odorant molecules (OM) and molecular receptors is inherently dynamic. Indeed, DM systems (refer to as dimer system) continually interconvert among multiple conformations, i.e., jumping between minima on the potential energy surface (PES). Previous studies have shown that conformations of large molecules in both gas-phase [42] and deposited on graphene nanoribbon [43] can exhibit comparable quantum-mechanical (QM) properties, raising the question of whether such effects also occur in these DM systems. Therefore, instead of considering only the most stable conformer for each DM configuration, as is common in many DFT studies, we sampled a broader ensemble of low-energy conformers and adsorbed them onto graphene. This procedure expands the MORE-Q dataset [23] into MORE-QX and increases the number of CPLX systems from 1, 836 to 10, 441 (see Figs. 1a–c). Accordingly, the same set of QM properties computed for the CPLX systems in MORE-Q was also calculated for the additional DM conformations. Note that the number of sampled conformers is adaptively adjusted to the morphological complexity of the DM system to ensure robust sampling. This means that systems with more flexible morphologies will yield a larger number of sampled conformers. On average, we considered six conformers per DM system. The initial set of 83,916 dimer configurations (50 per combination) was generated using the automated Interaction Site Screening (aISS) package [44]. Previous benchmarking on representative intermolecular complexes showed that aISS produces structures comparable in quality to those obtained with the widely used CREST code [44, 45], while requiring substantially lower computational cost. This improvement in efficiency mainly arises from the demanding metadynamics-based conformational search employed in the CREST workflow, which makes it less practical for the efficient exploration of a large number of non-covalent complexes. After generating dimer configurations with aISS, we then remove redundant conformers within each BOV–receptor combination by clustering the structures by hierarchical clustering based on the user-defined pairwise root-mean-square deviation (RMSD) similarity distance. A larger cutoff distance leads to fewer clusters and vice versa. To balance computational cost and configurational diversity, we examined the number of clusters obtained for each combination as a function of the similarity cutoff and ultimately selected a value of 1.5 Å. This strategy enables flexible conformer selection: combinations with more complex configurational landscapes retain more conformers, whereas simpler combinations retain fewer. As a result, 10,411 conformers were obtained for subsequent deposition on the graphene surface.

### MORE-QX property space

Similar to the MORE-Q dataset [23], the quantum-mechanical (QM) properties of BOVs, molecular receptors, and their dimer conformations contained in MORE-QX dataset were obtained both at GFN2-xTB+D4 level and PBE+D3 with def2-TZVPP basis set using xTB (version 6.6.0) [46] and ORCA (version 5.0.3) [47] packages, respectively. The dimer interaction energy $E_\mathrm{int}$ is defined as the total energy of the dimer conformation minus the energies of the individual constituents, i.e.,

1 $$\begin{aligned} E_\mathrm{int} = E_\mathrm{DM} - E_\mathrm{REC} - E_\mathrm{OM}. \end{aligned}$$

The BOV-receptor-graphene complex (CPLX) systems underwent geometry optimization using the DFTB+ [48] package, employing the GFN2-xTB Hamiltonian with D4 dispersion correction. While optimizing the structures, we fixed the atomic positions in the graphene layer, as the adsorption of the OM molecules will not significantly affect the geometry of graphene, and the electrode is restricting the deformation degree of the graphene for a chemiresistive sensing device. To create the SUB system (or REC-graphene system), we removed the BOV molecule from the CPLX system and did not optimize the structures in order to investigate the pure electronic effect of the binding features.

MORE-QX provides extensive sets of QM global and local properties (up to 39) for single BOV/receptor molecules (MORE-QX-G1), BOV-receptor molecular dimers (MORE-QX-G2), and complex systems (MORE-QX-G3). The MORE-QX-G1 subset contains QM property data for 102 BOV molecules and 18 molecular receptors. Among the 39 molecular and atomic properties, we computed the D3 energy, dipole moment, polarizability, and Mulliken charges. The MORE-QX-G2 subset is built on the geometries from MORE-QX-G1 via the search for molecular docking conformations using BOV molecules and receptors. Accordingly, MORE-QX-G2 contains QM property data for 23,838 dimer conformations at the GFN2-xTB+D4 level and for 10,411 dimers with the lowest binding energies at the PBE+D3 level (see the property list in Tables S2 and S3 of the SI). The MORE-QX-G3 subset contains 10,411 selected dimers from MORE-QX-G2 on graphene surface. Consequently, MORE-Q-G3 includes QM property data at the PBE+D3 level for both the CPLX and SUB systems, as well as binding features that account for property changes in single systems induced by BOV molecule adsorption. The expansion including GFN-xTB+D4 geometry relaxation and DFT calculation took $\sim 25$ Mio CPUhs.

To measure the correlation between QM properties in MORE-QX, we have used the Spearman correlation factor, which is computed as follow:

2 $$\begin{aligned} |\rho _s| = |1 - \frac{6\sum ^n_{i=1}d^2_i}{n(n^2-1)}|, \end{aligned}$$

where each paired observation $(X_i,Y_i)$’s respective ranks denotes $R(X_i)$ and $R(Y_i)$, and then the $d_i$ is defined as $d_i = R(X_i) - R(Y_i)$. Spearman is chosen owing to its robustness against outliers and the enhanced non-linear capturing ability compared to the counterpart Pearson correlation.

#### Binding feature calculation

As stated above, the combination of GFN2-xTB for molecular structure generation and PBE+D3 for property calculations provides a suitable and balanced strategy for generating a comprehensive dataset of molecule-substrate systems. The reliability of GFN2-xTB for generating molecular structures has already been demonstrated in several dataset-generation workflows (e.g., QCML [49], QMugs [50]). Similarly, the PBE functional, as implemented in the Vienna ab initio simulation package (VASP [51, 52], version 6.3.1), has been extensively used to generate benchmark materials datasets such as Alexandria [53], OMat24 [54], and MPtrj [55]. The PBE functional has also been employed to investigate diverse surface-induced properties in inorganic and organic–inorganic systems, such as work-function changes [56–58]. This widespread use indicates that PBE remains a good compromise between accuracy and computational efficiency for calculating properties across a large number of material systems. We further include the D3 van der Waals dispersion correction with PBE to improve the description of long-range interactions, which are particularly important when molecular dimers are deposited on substrates. A comparison with the more accurate PBE0+D3 functional shows that PBE+D3 and PBE0+D3 produce similar trends for most electronic properties (see Fig. S11 of the SI). However, the main difference lies in the computational cost, which increases significantly for complex systems containing approximately 300 atoms. Under the same computational setup, the average PBE0+D3 calculation time was approximately twelve times longer than that of PBE+D3 when using 256 cores of an Intel Xeon Platinum 8470 processor. This would represent a substantial increase in computational resources for calculating the binding features of the 10,411 complex systems considered in MORE-QX. Therefore, electronic-structure calculations of the SUB and CPLX systems were conducted at tightly converged PBE+D3 theory level by VASP package. The energy cutoff for the plane-wave basis set and the SCF convergence threshold were set to 600 and $1\cdot 10^{-4}$ eV, respectively. And all simulations were conducted at Gamma point. The dipole correction along the slab direction (50.68Å) was switched on to obtain flat electrostatic potential.

To compute the binding features, we carried out different type of DFT calculations. The adsorption energy ($E_{\mathrm{ads}}$) was obtained from total energies of single-point calculations and is defined as follows:

3 $$\begin{aligned} E_{\mathrm{ads}} = E_{\mathrm{CPLX}} - E_{\mathrm{SUB}} - E_{\mathrm{OM}}. \end{aligned}$$

Whereas, the work function change ($\Delta \phi$) is defined as the difference between the work function ($\phi$) after and before the BOV adsorption, i.e., $\phi$ for CPLX and SUB systems:

4 $$\begin{aligned} \Delta \phi = \phi _{\mathrm{CPLX}} - \phi _{\mathrm{SUB}}. \end{aligned}$$

Here, $\phi$ of each system was calculated using:

5 $$\begin{aligned} \phi = E_{\mathrm{V}} - E_{\mathrm{F}}, \end{aligned}$$

where $E_\mathrm{F}$ is the Fermi level and $E_\mathrm{V}$ is the vacuum energy. $E_\mathrm{V}$ is obtained by analyzing the flattened region of the electrostatic potential *P*(*z*) along the slab direction. *P*(*z*) is computed by the following equation:

6 $$\begin{aligned} P(z) = \int n(z) dz, \end{aligned}$$

where the planar averaged charge density *n*(*z*) is defined as:

7 $$\begin{aligned} n(z) = 1/A \iint n(x,y,z) dxdy \end{aligned}$$

Finally, the charge transfer $\Delta Q$ is computed as the total Bader charge [59] transferring between the BOV molecule and SUB system.

### MORE-ML framework

We designed the **M**olecular **O**lfactorial **R**eceptor **E**ngineering by **M**achine **L**earning (**MORE-ML**) framework to simultaneously perform binding feature regression and model explanation tasks, as illustrated in Fig. 3a. Among the spectrum of ML algorithms, linear models offer the highest explainability but lack sufficient capacity, whereas neural networks provide exceptional representational power yet suffer from nascent explainability [60]. To strike a balance between predictive performance and transparency, we employ tree-based models, which deliver both robust accuracy and an inherently interpretable decision process via hierarchical splitting [61]. Moreover, when integrated with explainable artificial intelligence (XAI) tools–e.g., **SH**apley **A**dditive ex**P**lanations (SHAP) [41]–these models not only yield precise predictions of binding features but also facilitate the extraction of underlying physical insights [62].

Building on the defined ML tasks, we now describe the training procedure for a single ML model, as depicted in Fig. 3b. In our initial training loops, we identified some systems in which the dominant interactions occurred between the OM and the graphene surface rather than with the receptor. By projecting the data into UMAP space and clustering based on SHAP values (for better cluster forming [63, 64]), we uncovered a distinct cluster corresponding to these outliers. We subsequently removed all 932 systems, as they lie outside the scope of DM pair design and would otherwise impair the effectiveness of our model (see more details in Fig. S4 of the SI).

The remaining data points are then split into training and test sets via farthest-point sampling (FPS) in the binding-feature t-SNE space, since t-SNE captures nonlinear relationships and clusters systems with similar binding mechanisms—preserving local consistency better than alternatives such as PCA or UMAP and homogeneous sampling in this space minimizes distributional divergence between the two sets. The dataset was partitioned into training and test sets at a fixed ratio of $9\!:\!1$. The corresponding learning curves are shown in Fig. S5. This fixed test set is used to benchmark both intermediate models and the final model throughout the entire training process. Then we conducted 100 iterations of Bayesian optimization (BO) to identify optimal hyperparameters, using the mean root-mean-square error (mRMSE) from 10-fold cross-validation at each BO iteration as the objective. The best-found hyperparameters were then applied to retrain the models on the training set, and final performance was evaluated on the fixed test set.

### Explainability strategy for tree-based regression models

#### Post-hoc explanation

In this work, we employ **SH**apley **A**dditive ex**P**lanations (SHAP) to interpret ML regression models developed for predicting binding features in a post-hoc manner. SHAP is a game-theoretic framework for explaining ML model outputs, grounded in cooperative game theory and based on Shapley values, which quantify how each input feature influences the deviation of an individual prediction from the expected/average output of the model. Consequently, this method allows for a more transparent interpretation of the learned correlations, highlighting the relative importance of features and how they interact to affect the predicted outcomes. SHAP converts the value of feature *j* to the SHAP value $\phi _j$ by considering its margin contribution towards the model *f* output, and hence the SHAP value of feature *j* is defined as:

8 $$\begin{aligned} \phi _j(f) = \sum _{S\subseteq N \backslash \{j\}} \frac{|S|!(|N|-|S|-1)!}{|N|!} [f(S \cup \{j\}) - f\{S\}], \end{aligned}$$

where *S* stands for feature subset without feature *j*, *N* is the total feature set, and *f* is the ML model. This equation defines the SHAP value as the sum of feature *j*’s marginal contributions across every subset *S*, each term weighted by the probability that exactly those features in *S* appear before *j* in all ordering combinations of all features.

#### Intrinsic explainability

In the same context, we also use the intrinsic explainability of decision-tree–based models, which formulate predictions as a nested rule structure. Starting from the root node, the model recursively subdivides the feature space by applying feature-dependent thresholding conditions (e.g., border values in CatBoost), producing a hierarchy of progressively constrained decision subspaces. The partitioning process terminates at leaf nodes, each associated with a fixed prediction value or a set of distributional parameters. The prediction mechanism for any leaf can be explicitly recovered by back-tracking along its unique partition path, yielding an interpretable representation of the model as a piecewise-constant function over disjoint regions of the input space. Therefore, we further analyze two complementary intrinsic metrics of the tree-based models to support and validate the findings obtained through the SHAP method: (1) node splitting frequency and (2) split threshold values. Specifically, we quantify how often each feature is used for node splitting across different tree depths to assess its global importance. In addition, we examine the range of threshold values associated with each feature to better understand how variations in feature values influence the model’s decision-making process and overall importance.

## Results and discussion

**Fig. 2:**
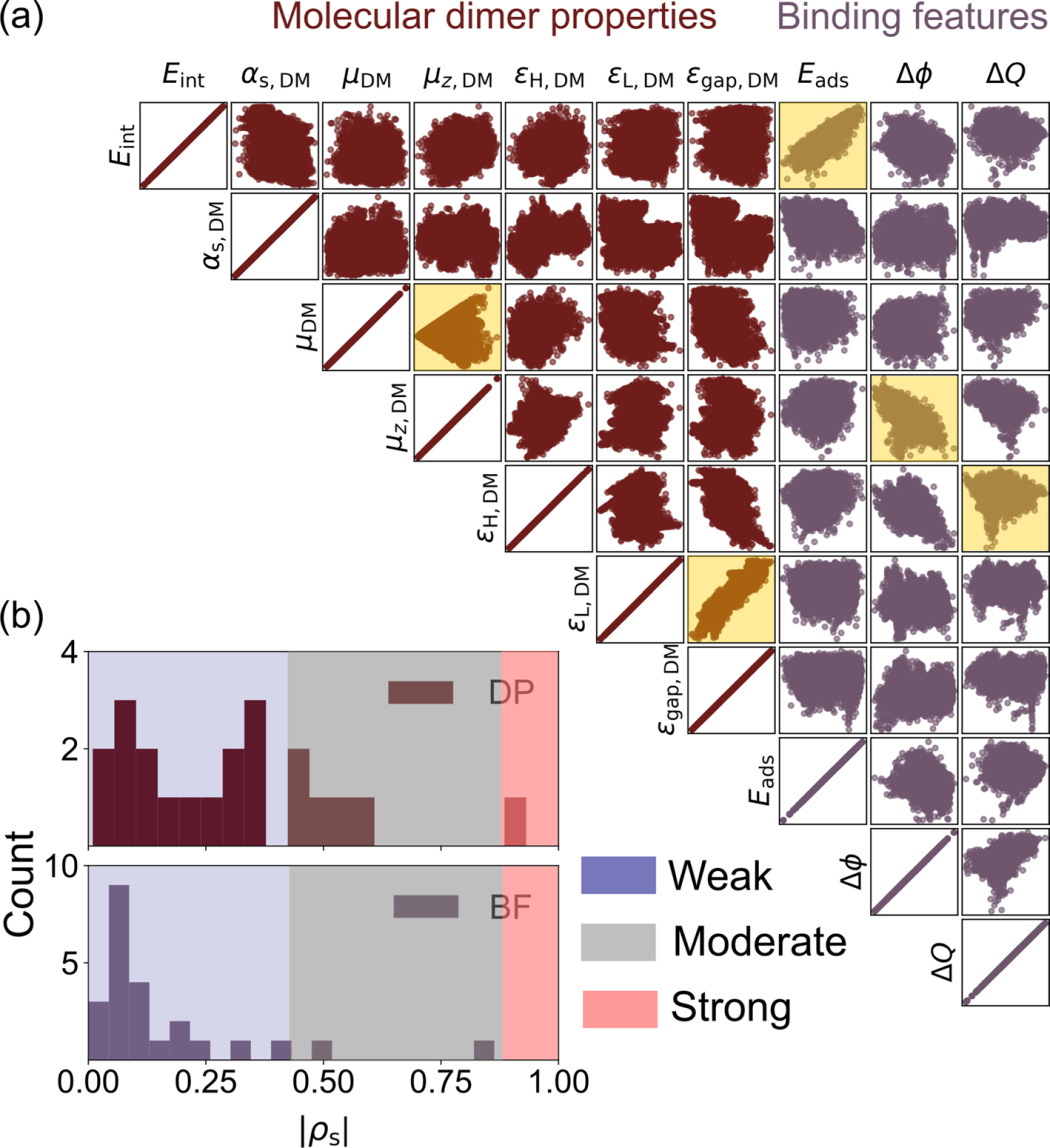
**a** Two-dimensional (2D) projections of the high-dimensional property space spanned by MORE-QX dataset. We show the correlation plots for seven dimer properties (DP, brown) and three binding features (BF, purple) from MORE-QX. The detailed description of the properties can be viewed in Table 1. Some interesting projections are marked by yellow frames and discussed in the manuscript. **b** The count measurement of absolute value of Spearman correlation coefficient $|\rho _s|$ for DP (upper panel) and BF (lower panel) 2D projections. The $|\rho _s|$ values result in three distinct clusters: weakly correlated $|\rho _s| \le 0.5$, moderately correlated $0.5 < |\rho _s| \le 0.8$, and strongly correlated $|\rho _s| > 0.8$ covering by blue, gray, red frames, respectively

We first analyze two-dimensional (2D) projections of the high-dimensional property space spanned by CPLX systems in MORE-QX. In Fig. 1d, we illustrate the property space defined by the work function change ($\Delta \phi$) and the dimer interaction energy ($E_{\mathrm{int}}$), i.e., $\left( \Delta \phi , E_{\mathrm{int}} \right)$. Overall, one can see a lack of correlation between both properties, which indicates a degree of flexibility when searching for dimer conformations with a given pair of $\left( \Delta \phi , E_{\mathrm{int}} \right)$ values. To understand better the influence of conformational sampling, two example configurations were selected, see rectangles in Fig. 1d. In the red rectangle, $\Delta \phi$ is also uncorrelated with $E_{\mathrm{int}}$ and displays a large variation in magnitude with respect to the value corresponding to the most stable conformation, from 0.0 to 0.4 $\mathrm{eV}$. This change is also much larger compared to $E_{\mathrm{int}}$ that only decreases from $-0.8$ to $-0.75$ $\mathrm{eV}$ (i.e., $\sim 0.05\,\mathrm{eV}$). Similarly, in the second set of studied conformations (enclosed by the rectangle blue), $E_{\mathrm{int}}$ is reduced because the OM molecule is changed by a smaller one, but $\Delta \phi$ still covers a larger property range ($\sim 0.25\,\mathrm{eV}$) This flexibility persists across the entire $\left( \Delta \phi , E_{\mathrm{int}} \right)$ property space, independent of the chosen DM configuration, underscoring the complexity of inferring binding features from QM properties of DM conformations. Moreover, this result already indicates the challenge in determining simple physical and chemical rules for the simultaneous optimization of properties in the MORE-QX property space (*vide infra*). Nevertheless, Fig. 1d conveys another important message for designing artificial olfactory systems: given a fixed $E_{\mathrm{int}}$, we might be able to find multiple CPLX systems with a desired $\Delta \phi$ value within a large range. Inversely, it is also possible to find different DM configurations with a desired $E_{\mathrm{int}}$ value in a large $\Delta \phi$ range. These initial observations provide the first evidence of an intrinsic “Freedom of design” in the MORE-QX property space [40], which will be discussed in the context of the binding feature space in the next section (see Fig. 2). Additional property distributions representing the effect of conformational sampling can be found in Fig. S1 of the Supplementary Information (SI).

**Table 1:** List of relevant physicochemical properties for BOV-receptor (dimer systerm, DM) and BOV-receptor-graphene (complex system, CPLX) interaction

#	Property	Symbol	Unit
1	Interaction energy	$E_{\mathrm{int}}$	eV
2	Isotropic molecular polarizability	$\alpha _\mathrm{s,DM}$	$a_0^{3}$
3	Scalar dipole moment	$\mu _\mathrm{DM}$	D
4	Dipole moment component along slab (*z*) direction	$\mu _{z,\mathrm DM}$	D
5	HOMO energy	$\epsilon _\mathrm{H,DM}$	eV
6	LUMO energy	$\epsilon _\mathrm{L,DM}$	eV
7	HOMO-LUMO gap	$\epsilon _\mathrm{gap}$	eV
8	Adsorption energy	$E _\mathrm{ads}$	eV
9	Work function change	$\Delta \phi$	eV
10	Charge transfer	$\Delta Q$	e

Each property presents a name, symbol, unit. $a_0$ and D refer to the atomic unit of Bohr radius and Debye

### “Freedom of design” in the MORE-QX property space

To gain a deeper understanding of the relationship between the QM properties of the building blocks and the resulting binding features (BFs), we examined selected pairwise correlations within the high-dimensional property space spanned by MORE-QX. Specifically, we analyzed correlations between the properties of DM systems and the associated BFs (see the full property list in Table 1). DM properties were selected because of their strong involvement in physicochemical effects arising from molecule–surface interactions, such as orbital hybridization, polarization effects, charge density redistribution, and charge transfer, which ultimately influence the binding features [65]. Overall, Fig. 2a shows that nearly all of the 45 unique pairwise projections (i.e., 2D correlation plots) resemble structureless “blobs”, indicating that most of these QM properties are effectively uncorrelated. To quantify the degree of correlation, we computed the absolute value of the Spearman correlation coefficient, $|\rho _s|$ (see Eq. 2). The distributions of $|\rho _s|$ for DM properties and BFs are shown in the upper and lower panels of Fig. 2b, respectively, where the pairwise correlations are categorized according to their $|\rho _s|$ values. Properties are considered strongly correlated if $|\rho _s| > 0.8$, moderately correlated if $0.5 < |\rho _s| \le 0.8$, and weakly correlated if $|\rho _s| \le 0.5$. Accordingly, among the DM properties, 1 out of 21 pairwise correlations ($\approx 4.8\%$) is strongly correlated, 4 out of 21 ($\approx 19\%$) are moderately correlated, and the remaining 16 ($\approx 76\%$) are weakly correlated. In contrast, none of the 24 correlations associated with BFs are strongly correlated; 2 out of 24 ($\approx 8\%$) exhibit moderate correlation, while the remaining 22 ($\approx 92\%$) are weakly correlated. This comparison demonstrates that correlations are generally weak for both DM properties and BFs, with correlations among BFs being even weaker than those among DM properties. This behavior reflects a more intricate and nontrivial interplay of interatomic interactions in OM–REC–graphene (CPLX) systems compared to single dimers (OM-REC systems).

Among the 2D property spaces analyzed, a few cases of interest exhibit moderate to strong correlations (highlighted by yellow frames in Fig. 2a). For example, the HOMO-LUMO gap ($\epsilon _{\mathrm{gap,DM}}$) of DM systems shows a more linear correlation with the LUMO energy ($\epsilon _{\mathrm{L,DM}}$) than with the HOMO energy ($\epsilon _{\mathrm{H,DM}}$). This observation implies that $\epsilon _{\mathrm{H,DM}}$ can be used to distinguish DM systems with similar $\epsilon _{\mathrm{gap,DM}}$, which is an important requirement for constructing efficient electronic descriptors. Regarding correlations with BFs, the interaction energy ($E_{\mathrm{int}}$) of DM systems and the corresponding adsorption energy ($E_{\mathrm{ads}}$) exhibit a strong correlation, with $|\rho _s| = 0.86$. This result suggests that the interaction mechanism between OM and REC systems can be transferred to CPLX systems to describe trends in $E_{\mathrm{ads}}$. However, the correlation is not fully linear, indicating that fluctuations arise from geometry and charge-distribution changes induced by surface interactions. Another relevant BF is the adsorbate-induced charge transfer ($\Delta Q$), which is commonly interpreted within the orbital-mixing theory that describes the alignment between the substrate Fermi level (the DM system in this work) and the frontier orbital energies of the adsorbate [66]. By computing $|\rho _s|$ between $\Delta Q$ and the orbital energies of DM systems, we find that both HOMO and LUMO energies are only weakly correlated with $\Delta Q$, with $|\rho _s| = 0.02$ and $|\rho _s| = 0.11$, respectively. Similarly, orbital energies associated to OM systems are also uncorrelated with $\Delta Q$, yielding $|\rho _s| < 0.3$. This lack of correlation reveals the complexity of using orbital energies alone to define design principles for tuning $\Delta Q$. At the same time, it reflects a certain “freedom of design” within the binding feature space, enabling the identification of DM systems with targeted orbital energies that can serve as components of electronic descriptors for BF prediction (*vide infra*).

In our correlation analysis with the work function change ($\Delta \phi$), we found that the z-component of the dipole moment in the DM ($\mu _{\mathrm{z,DM}}$) and OM ($\mu _{\mathrm{z,OM}}$) systems shows a moderate correlation with $\Delta \phi$, with $|\rho _s| = 0.51$ and $|\rho _s| = 0.68$, respectively. As discussed in our previous work [67–69], $\Delta \phi$ follows the Helmholtz relation:

9 $$\begin{aligned} \Delta \phi = - e/\varepsilon _{\mathrm{0}}\cdot \Delta P_{\mathrm{tot}}, \end{aligned}$$

where surface dipole moment change ($\Delta P_{\mathrm{tot}}$) could be split into several components as,

10 $$\begin{aligned} \Delta \phi = - e/\varepsilon _\mathrm{0} \cdot (\Delta p_{\mathrm{cplx}} + p_{\mathrm{a}} + p_{\mathrm{s}} - p_0 ), \end{aligned}$$

where the components $\Delta p_{\mathrm{cplx}}$, $p_{\mathrm{a}}$, and $p_{\mathrm{s}} - p_0$ denote the adsorbate-induced surface dipole moment change by spatial charge redistribution, adsorbate dipole moment, and surface deformation, respectively. $\mu _{\mathrm{z,DM}}$ inherently contains information related to $\mu _{\mathrm{z,OM}}$, which is tightly associated with the $p_a$ term and yields a moderate correlation ($|\rho _s| = 0.51$). However, other contributions—particularly $\Delta p_{\mathrm{cplx}}$, which describes spatial charge redistribution—are poorly captured by $\mu _{\mathrm{z,DM}}$ or by any other DM property e.g., polarizability $\alpha _\mathrm{S,DM}$, as evidenced by the very low correlation ($|\rho _s| = 0.07$ between $\Delta \phi$ and $\alpha _\mathrm{S,DM}$). These findings highlight both the intrinsic complexity of $\Delta \phi$ and the insufficiency of current physicochemical heuristics for tailoring it. While the weak-to-moderate correlations between DM properties and BFs provide some theoretical guidance based on physicochemical intuition, no clear patterns emerge to navigate the binding feature space. Moreover, there is little correlation among the BFs themselves. For example, $E_\mathrm{ads}$ is only weakly correlated with $\Delta \phi$ ($|\rho _s| = 0.14$). Likewise, $\Delta Q$ shows a weak correlation with $E_\mathrm{ads}$ ($|\rho _s| = 0.05$) and a moderate correlation with $\Delta \phi$ ($|\rho _s| = 0.40$), the latter arising from spatial charge redistribution upon adsorption [67, 69]. Collectively, these observations indicate that only few constraints limit a DM system from simultaneously exhibiting any given pair of DM and BF properties considered in Fig. 2a, providing compelling evidence for the existence of a “freedom of design” in the binding feature space. Building on this concept, we also analyzed how the weak correlations among BFs enable the identification of DM conformations tailored to specific target properties (see the SI for details). Consequently, a large number of electronic features may serve as efficient molecular descriptors for BF prediction; however, owing to differences in correlation strength and underlying physicochemical insight, some descriptors are likely to be more relevant than others.

Notice that, even though Boltzmann-weighted properties could in principle be used to construct more accurate ensembles [70], we treat each low-energy dimer conformer equally in order to probe conformer-specific effects and to explore the potential energy surface more comprehensively than a static Boltzmann average would allow. Because the low-energy conformers have similar Boltzmann weights, weighting or direct averaging would obscure subtle inter-conformer differences. Since our primary goal is to examine how BFs vary across individual surface-bound dimers, we therefore do not apply Boltzmann weighting.

**Fig. 3:**
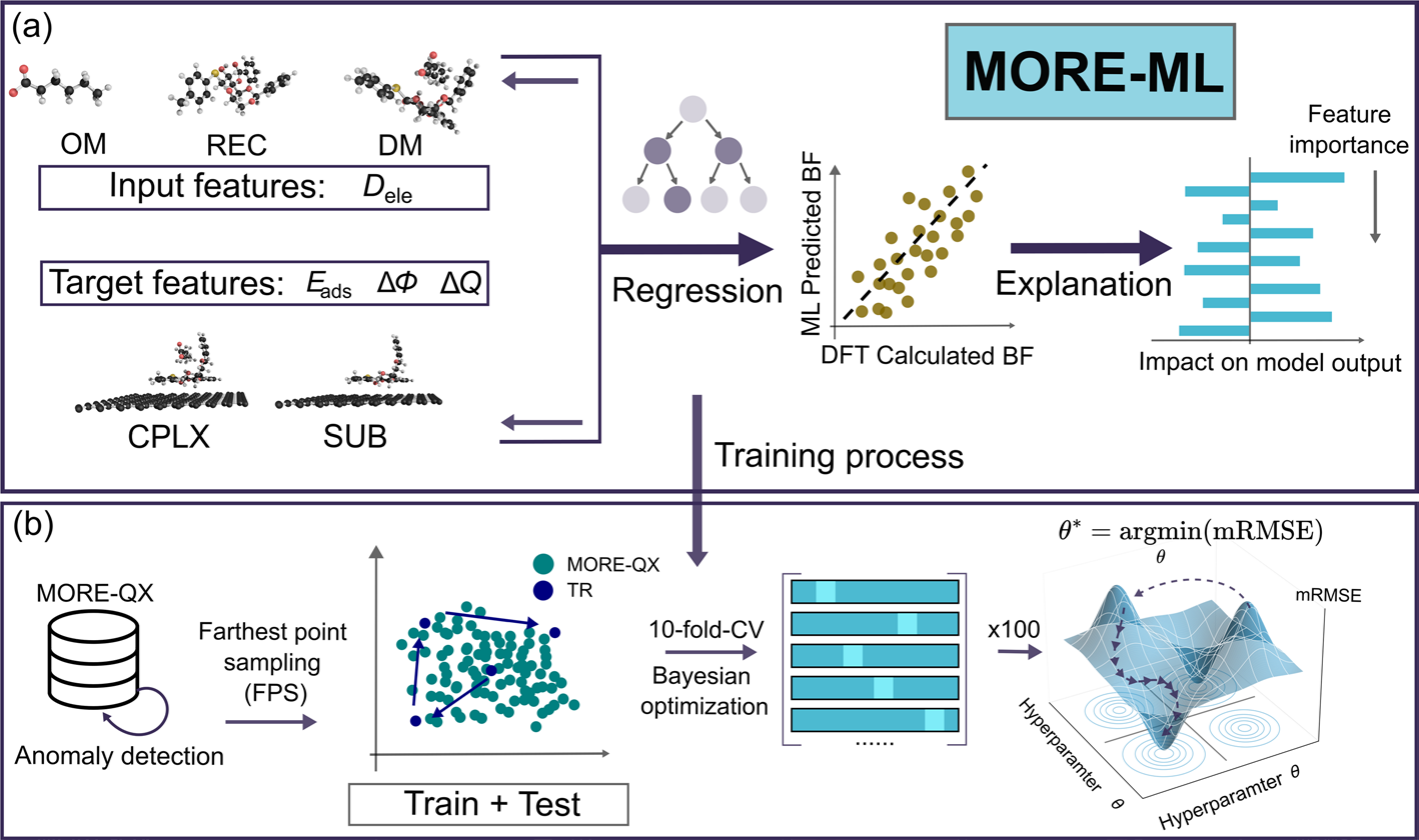
**a** Scheme of the MORE-ML framework, which stands for **M**olecular **O**lfactorial **R**eceptor **E**ngineering by **M**achine **L**earning, which integrates QM properties of molecular building blocks ($\mathrm D_{ele}$) with ML techniques for the prediction of binding features (BFs) such as $E_\mathrm{ads}$, $\Delta \phi$ and $\Delta Q$. MORE-ML framework aims at regression and model explanation tasks. **b** ML model training in MORE-ML starts with anomaly detection (see SI), followed by farthest point sampling (see Methods) to construct the training and test sets. Bayesian optimization with 100 iterations and 10-fold cross-validation on the training set is used for hyperparameter tuning. Final model performance is evaluated on the test set

### Navigating the binding feature space via machine learning

Although the lack of correlation among BFs provides a flexibility in designing CPLX systems with desired sensing-related properties, determining the relationship between BFs and electronic properties of molecular building blocks (OM, REC, and DM systems) is still challenging. To address this issue, we have implemented the machine learning (ML) framework MORE-ML (see Fig. 3), which aims at establishing a quantitative and explainable mapping between these property spaces by using ML regression techniques and explainable AI methods (see Methods). To identify the most suitable regression models for BF prediction, we benchmark the performance of several tree-based methods: random forest (RF) [71], gradient boosting decision trees (GB), XGBoost (XGB) [72], CatBoost (CAT) [73], and LightGBM (LGBM) [74]. The best-performing models will be subsequently analyzed using SHapley Additive exPlanations (SHAP) [41], an efficient explainable AI framework well suited to tree-based models. As a training strategy, we prioritized electronic-structure–derived descriptors ($D_\mathrm{ele}$), composed of QM properties of OM, REC, and DM systems, owing to their lightweight nature and clear physicochemical interpretability (see Table S3 in the SI). Moreover, inspired by the development of the QUED framework [21], we investigated whether model performance could be further improved by combining $D_\mathrm{ele}$ with geometrical descriptors $D_\mathrm{geo}$ (e.g., Bag-of-Bonds [75], SOAP [76], and MACE [77]) and the corresponding Mulliken atomic charges *q* ($D_q$). However, as shown in Figs. S7 and S8 of the SI, the inclusion of these additional descriptors did not improve the performance of the ML models. This effect may be attributed to the fact that certain electronic features of DM systems already encode relevant geometric information. For example, the dipole moment, $\mu _\mathrm{DM}$, is the most sensitive to structural variations across different conformations, with changes of up to 80% (see Fig. S12 of the SI). The HOMO–LUMO gap, $\epsilon _{gap}$, exhibits fluctuations of up to 20%, whereas the HOMO energy, $\epsilon _{H, DM}$, shows smaller variations of up to 8% across different DM configurations. Consequently, explicit geometric descriptors provide limited additional predictive value, as geometry-dependent information is already implicitly captured by the electronic descriptors. Regarding the $D_q$ descriptor, its limited predictive power may stem from the accuracy of Mulliken charges. However, addressing this limitation would require adopting more robust charge partitioning schemes (e.g., CM5 or Hirshfeld) implemented in alternative DFT packages, or developing dedicated ML models for charge prediction, which is beyond the scope of the present work. Accordingly, we performed a more in-depth analysis of ML model accuracy using only $D_\mathrm{ele}$.

**Fig. 4:**
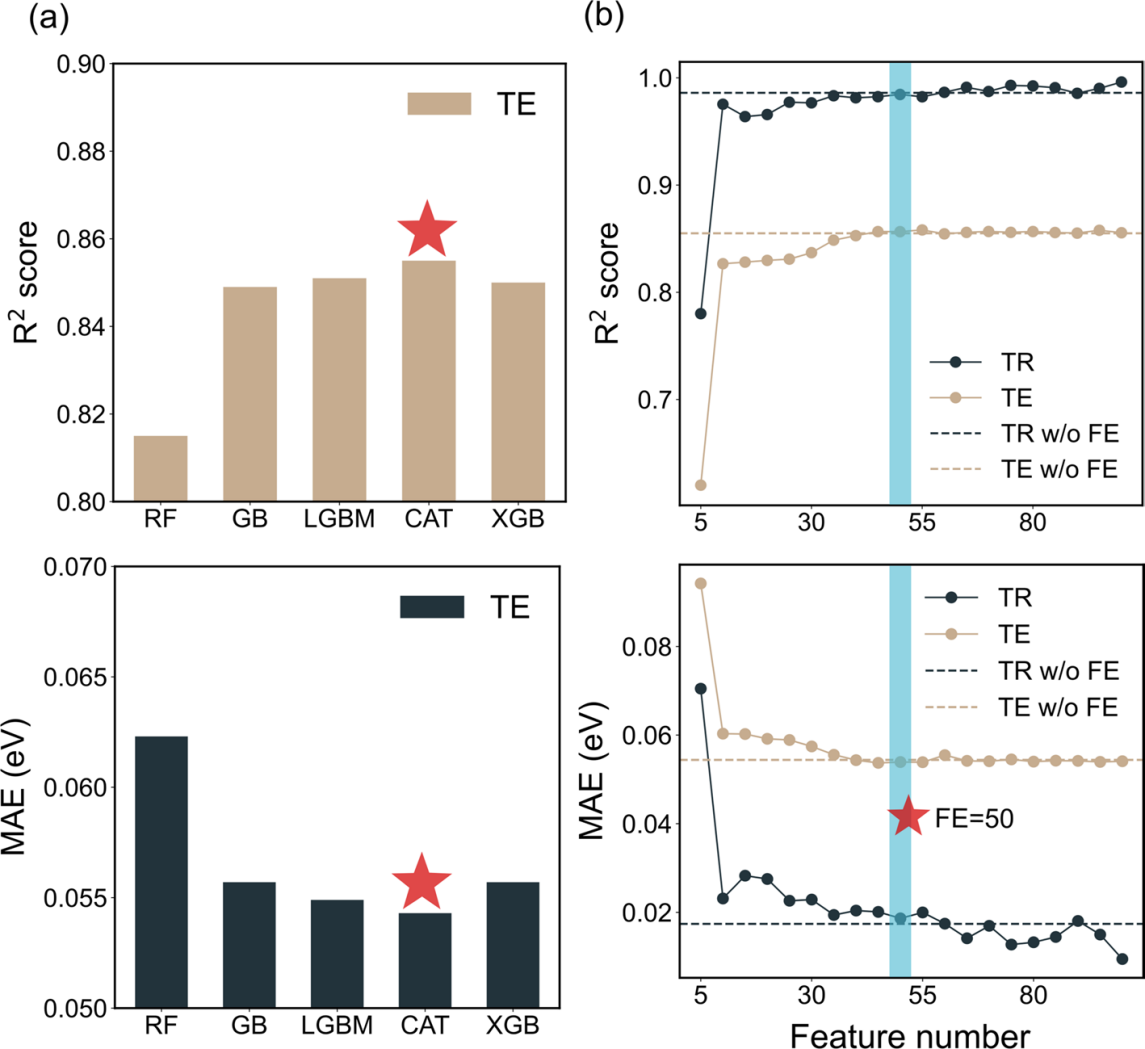
Model benchmarking and feature engineering for the prediction of adsorption energy ($E_\mathrm{ads}$). **a** Coefficient of determination ($R^2$) and mean absolute error (MAE) evaluated on the test (TE) set for tree-based models: random forest (RF), gradient boosting decision tree (GB), LightGBM (LGBM), CatBoost (CAT), and XGBoost (XGB). The best-performing model is indicated by red stars. **b** Evolution of $R^2$ (upper panel) and MAE (lower panel) during feature engineering (FE) of the CatBoost model for predicting $E_\mathrm{ads}$. The number of features is increased incrementally in steps of five, ranked by SHAP analysis (see Methods). Model performance is shown for the training (TR, black) and test (TE, brown) sets. Dashed lines indicate performance obtained using the full set of QM properties as features

Fig. 4a shows the coefficient of determination ($R^2$) and mean absolute error (MAE) for predicting the adsorption energy, $E_{\mathrm{ads}}$, using the full $D_\mathrm{ele}$ descriptor (130 features). The MORE-QX dataset was partitioned into training (TR) and test (TE) sets using a fixed 9:1 ratio. Additional details on the dataset splitting procedure and the selection of training samples are provided in the Methods section. By comparing the results obtained for $E_{\mathrm{ads}}$ with those corresponding to other binding features (see Fig. S7 in the SI), we find that all benchmarked methods exhibit similar performance trends across features. Consequently, $E_{\mathrm{ads}}$ is used here as a representative case. Among the tree-based methods, RF performs the worst in the TE set, yielding an $R^2$ of 0.818 and an MAE of 0.062 eV. This suggests that gradient-boosting approaches outperform RF’s bagging strategy in capturing latent correlations between $D_\mathrm{ele}$ and the binding features. A likely explanation is that, in boosting, each successive tree corrects the residuals of its predecessor, whereas RF relies on an ensemble of independent trees. Within the gradient-boosting family, CAT achieves the best performance, with an $R^2$ of 0.86 and an MAE of 0.058 eV. This advantage likely arises from the use of oblivious (symmetric) trees, in which all nodes at a given depth split on the same feature. Such a structure imposes strong regularization on tree complexity, thereby improving generalization in binding feature prediction. As a result, we adopt CAT as the final ML regression model for subsequent analyses.

Then, we focus on selecting the most informative subset of QM properties within $D_\mathrm{ele}$ to mitigate high dimensionality and reduce model noise. By identifying and removing redundant and highly correlated features, we aim to prevent overfitting and improve the generalizability of the ML model. To this end, we employed an iterative SHAP-driven feature selection procedure using the CAT models. At each iteration, the model is retrained with a reduced subset of the full $D_\mathrm{ele}$, consisting of the top-ranked features according to SHAP importance. The number of selected features was gradually increased from 5 to 105 in increments of 5, and model performance was evaluated at each stage. The resulting learning curves for the $R^2$ and MAE metrics are shown in Fig. 4b. Based on the results for the TR and TE sets, the feature learning behavior can be divided into growing and saturated regimes. In the small-$D_\mathrm{ele}$ regime, performance improves gradually but remains inferior to that achieved with the full descriptor set (see dashed lines), indicating that an insufficient number of QM properties is available to accurately capture the adsorption mechanism. Once the size of $D_\mathrm{ele}$ exceeds a critical threshold, the performance curves begin to plateau: the TE scores no longer improve, while the TR scores show only minor fluctuations. This behavior indicates that additional features do not further enhance the model’s understanding of the adsorption mechanism, suggesting the existence of an optimal QM subset that balances accuracy and efficiency. Based on this exhaustive analysis, we selected the top 50 electronic features (star-labeled) as the effective descriptor set for $E_{\mathrm{ads}}$. Using the same procedure, the top 60 and top 80 features were selected for $\Delta \phi$ and $\Delta Q$, respectively (see Fig. S7 in the SI).

**Fig. 5:**
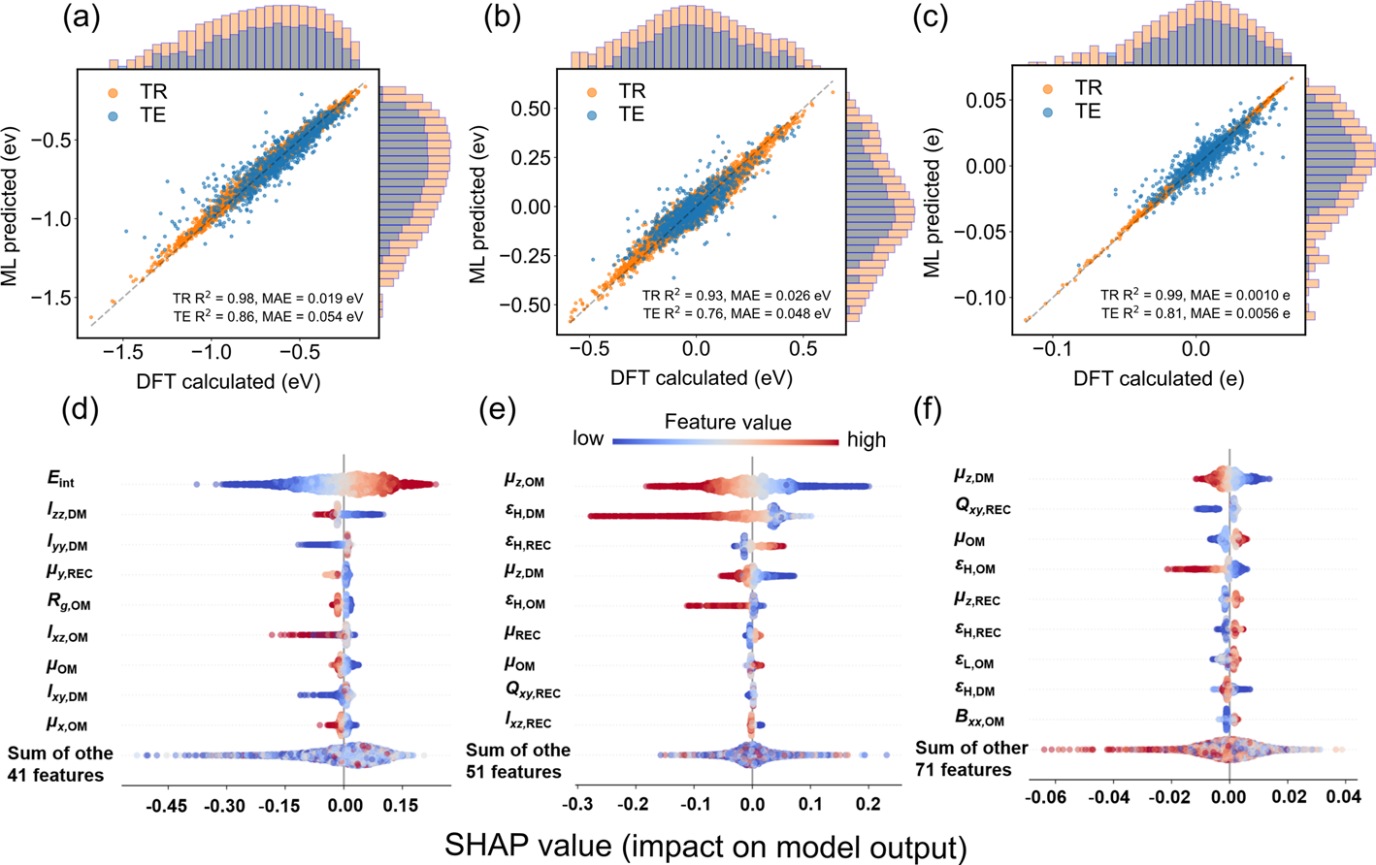
Correlation plots between DFT calculated and ML predicted values are shown for the best-performing models used to predict **a** $E_\mathrm{ads}$, **b** $\Delta \phi$, and **c** $\Delta Q$. Orange and blue bars/points represent the training (TR) and test (TE) sets, respectively. The lateral panels display the distributions for each binding feature. **d**–-**f** Show the corresponding SHAP beeswarm plots (see Methods) for **d** $E_\mathrm{ads}$, **e** $\Delta \phi$, and **f** $\Delta Q$. In each beeswarm plot, features are ranked in ascending order of importance from top to bottom, with SHAP values distributed around the zero baseline. Each point is colored according to the corresponding feature value. Only the top nine features are shown; the cumulative SHAP value of all remaining features is reported in the final column ($10^\mathrm{th}$ position)

Indeed, the final ML regression models for predicting $E_\mathrm{ads}$, $\Delta \phi$ and $\Delta Q$ were developed using the optimized subset of QM features and CAT method (see Fig. 5). To assess their overall learning capability, we first examine the $\mathrm R^2$ metric, which quantifies the variance between DFT-calculated and ML-predicted values. For TR set, $\mathrm R^2$ reaches 0.99 for both $E_\mathrm{ads}$ and $\Delta Q$, whereas a slightly lower value of 0.93 is obtained for $\Delta \phi$. This difference is reflected in the larger dispersion of the orange data points around the $y=x$ reference line (dashed). Considering the MAE metric, the corresponding values for TR set of $E_\mathrm{ads}$ and $\Delta Q$ are $0.017\,\mathrm eV$, and $0.001\,\mathrm e$, respectively, while $\Delta \phi$ exhibits a higher MAE of $0.026\,\mathrm eV$. Given the discrete nature of the binding feature space and the limited coverage of MORE-QX dataset, we further evaluate model performance using the relative error $\epsilon = \dfrac{|y_\mathrm{ML} -y_\mathrm{DFT}|}{\Delta y}\times 100$ with $y_\mathrm{ML}$ and $y_\mathrm{DFT}$ as the ML and DFT values of the property *y*. $\Delta y$ represents the extent of the property spectrum across the entire dataset. The resulting relative errors for $E_\mathrm{ads}$, $\Delta \phi$ and $\Delta Q$ are $1.7\%$, $2.6\%$ and $1\%$, respectively. These small values indicate that the models accurately reproduce the training data.

We next examine the generalization capability of the ML models by evaluating their performance on unseen systems considered in the TE set. As expected, model accuracy decreases relative to the TR set, yielding $\mathrm R^2$ values of 0.86, 0.76, and 0.81 for $E_\mathrm{ads}$, $\Delta \phi$, and $\Delta Q$, respectively. The relative errors also increases, but remain below $6\%$: $5.4\%$ for $E_\mathrm{ads}$, $4.8\%$ for $\Delta \phi$, and $5.6\%$ for $\Delta Q$. The moderate performance gap between the TR and TE sets indicates that the models retain high predictive accuracy for novel systems, underscoring their potential to generalize across a much larger configuration and conformational space. This conclusion is further supported by the close agreement between the distributions of predicted binding features for the TR and TE sets (see right panels in Figs. 5a–c). The complete set of evaluation metrics for each model is summarized in Table S6. To elucidate the slightly reduced accuracy of the model predicting $\Delta \phi$, we separately predicted $\phi$ values for both the complex ($\phi _\mathrm{CPLX}$) and the substrate ($\phi _\mathrm{SUB}$) systems using the same TR and TE sets. As shown in Fig. S10, the predictions for $\phi _\mathrm{CPLX}$ yield $\mathrm R^2 = 0.87$ and $\mathrm{MAE} = 0.045\, \mathrm eV$; while those for $\phi _\mathrm{SUB}$ achieve $\mathrm R^2 = 0.89$ and $\mathrm{MAE} = 0.029\, \mathrm eV$. Despite these favorable metrics, the parity plot for $\phi _\mathrm{SUB}$ exhibits an unexpected zigzag pattern in both the TR and TE sets, and the model fails to reproduce the bimodal distribution of $\phi _\mathrm{SUB}$. This shortcoming likely stems from the limited diversity of $\phi _\mathrm{SUB}$ values in the dataset: the QM descriptors of the building blocks, particularly those derived from the 18 REC structures, are insufficient to capture the subtle conformational variations that govern $\phi _\mathrm{SUB}$. Consequently, noise is introduced into the prediction of $\phi _\mathrm{SUB}$, even though $\phi _\mathrm{CPLX}$ is modeled accurately.

### AI-based explanation of binding feature predictive models

To better interpret the tree-based ML models developed for BF prediction, we performed an explainability analysis using both their intrinsic interpretability and SHAP method (see Methods). The beeswarm plots in Figs. 5d–f summarize the distribution of SHAP values for the most influential features in each prediction task. In these plots, features are ranked by importance from top to bottom, and their corresponding SHAP values are shown along the *x*-axis. Positive SHAP values indicate that a feature increases the predicted outcome, whereas negative values indicate a decrease. The color gradient encodes the feature magnitude, with red representing high values and blue representing low values.

The SHAP value distribution in Fig. 5d clearly shows that the dimer (DM) interaction energy, $E_\mathrm{int}$, plays the most dominant role in determining $E_\mathrm{ads}$. The color gradient indicates that smaller $E_\mathrm{int}$ values lead to smaller $E_\mathrm{ads}$ values and vice versa, since both quantities are negatives. This strong coupling between $E_\mathrm{int}$ and its SHAP value is reflected in the high Spearman correlation coefficient, $|\rho _s| = 0.86$, indicating that $E_\mathrm{int}$ serves as an effective descriptor for $E_\mathrm{ads}$ on the graphene surface. Although $E_\mathrm{int}$ contains the majority of the predictive information for $E_\mathrm{ads}$, the model still needs to account for a small residual difference between these two energetics to achieve higher accuracy. This difference is captured by morphological descriptors, such as the components of the inertia tensor (*I*) of the DM systems and the radius of gyration ($R_g$) of the OM system, highlighting that molecular structure also plays a critical role. Furthermore, the dipole moments ($\mu$) of OM and REC systems rank among the top ten features, indicating that charge redistribution is relevant for describing non-covalent interactions during adsorption. The SHAP values of these additional features are distributed much more narrowly than those of $E_\mathrm{int}$, which explains their lower overall importance. Consequently, these features-together with the remaining descriptors, primarily act as fine-tuning factors, capturing a small number of outliers and subtle corrections compared to the dominant contribution of $E_\mathrm{int}$.

**Fig. 6:**
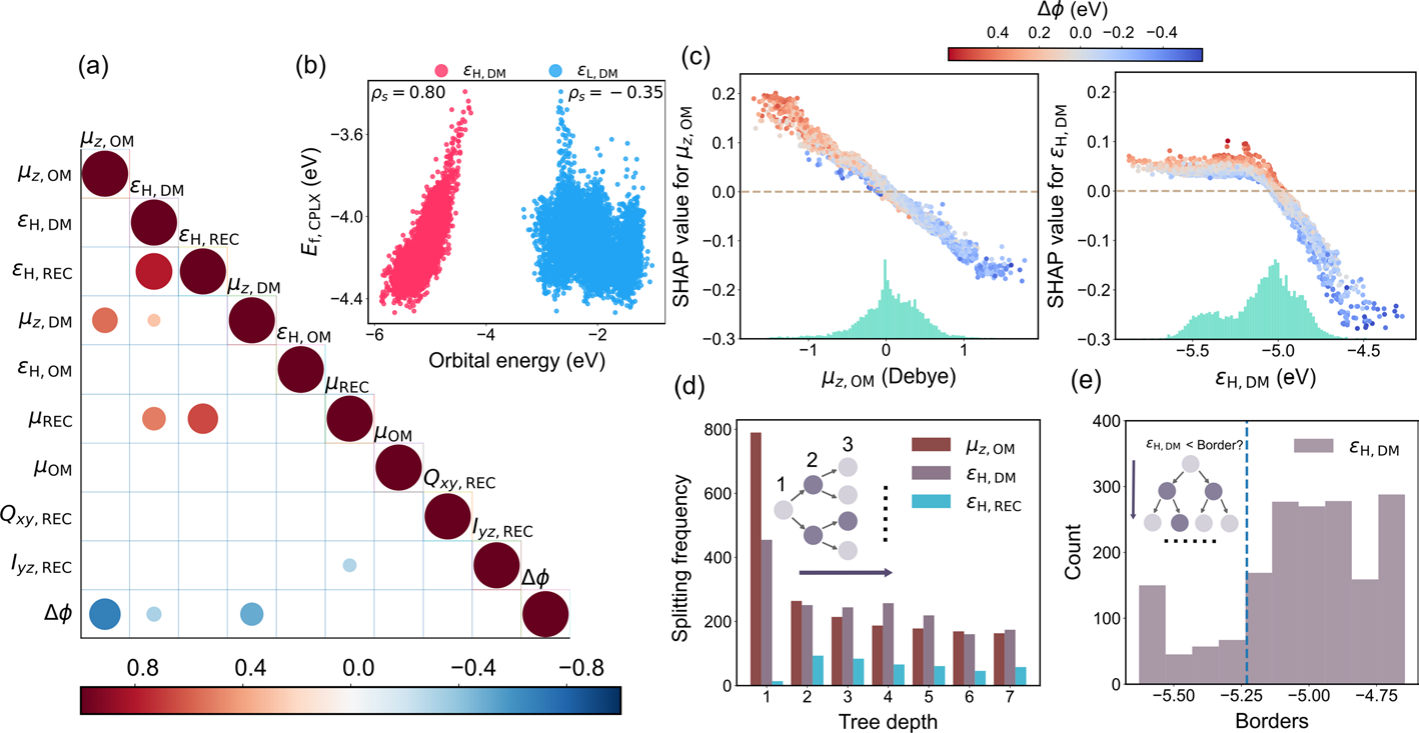
Explanation of the ML model for $\Delta \phi$ prediction. **a** Pairwise Spearman correlation coefficients $\rho _s$ between the top nine features and $\Delta \phi$. Circle size and color indicate the magnitude and sign of $\rho _s$, respectively. **b** Correlation plots between the Fermi level of the CPLX systems, $E_\text{f, CPLX}$, and the dimer HOMO $\epsilon _\mathrm{H,DM}$ (red) and LUMO $\epsilon _\mathrm{L,DM}$ (blue) energies. Corresponding $\rho _s$ values are shown in the plots. **c** SHAP value dependence plots for the two most important features: vertical dipole moment of OM $\mu _\mathrm{z,OM}$ (left panel) and dimer HOMO energy $\epsilon _\mathrm{H,DM}$ (right panel). SHAP values are shown as a function of the corresponding feature value; data points are colored by $\Delta \phi$, with feature value distributions shown along the *x*-axis. **d** Frequency of feature participation in node splits as a function of tree depth for the three most important features: $\mu _\mathrm{z,OM}$, $\epsilon _\mathrm{H,DM}$, and the receptor HOMO energies $\epsilon _\mathrm{H,REC}$. **e** Distribution of splitting frequencies as a function of the border values for $\epsilon _{\mathrm{H,DM}}$

A similar trend can be observed in the SHAP analysis for predicting $\Delta Q$ (see Fig. 5f). In contrast to the morphology-correlated binding feature $E_\mathrm{ads}$, $\Delta Q$ is primarily correlated with charge-related properties. In particular, the dipole and quadrupole moments emerge as the most relevant features, whereas molecular orbital energies appear lower in the ranking; e.g., $\epsilon _\mathrm{H,\mathrm OM}$ and $\epsilon _\mathrm{H,\mathrm REC}$ occupy the $4^\mathrm{th}$ and $6^\mathrm{th}$ positions, respectively. This indicates that several properties contribute synergistically to the prediction of $\Delta Q$, with no single dominant feature. Moreover, the SHAP analysis highlights the limited capability of a purely qualitative orbital-mixing description of charge transfer [66], as the frontier orbital energies are not among the dominant predictors. Unlike $E_\mathrm{ads}$ and $\Delta Q$, the prediction of $\Delta \phi$ is governed by two dominant features: the vertical dipole moment of the OM, $\mu _{z,\mathrm OM}$, and the HOMO energy of the DM system, $\epsilon _\mathrm{H,\mathrm DM}$, which exhibit a wide distribution in Fig. 5e. The importance of $\mu _{z,\mathrm OM}$ is readily explained by Eq. 10, since it directly contributes to the total change in the surface dipole moment. Interestingly, the SHAP distribution of $\epsilon _\mathrm{H,\mathrm OM}$ (ranked $5^\mathrm{th}$) shows a trend similar to that of $\epsilon _\mathrm{H,\mathrm DM}$, which is the second most important feature. A qualitative explanation for the high ranking of frontier orbital energies (HOMO/LUMO) is that $\Delta \phi$ partially originates from spatial charge redistribution. In this context, the HOMO and LUMO energies represent the primary donor and acceptor orbitals, respectively, thereby inducing charge-density changes on and near the associated atoms.

To gain further physical insight into the prediction of $\Delta \phi$, we first analyzed the Spearman correlation coefficient, $|\rho _s|$, between the top 10 QM features and $\Delta \phi$ (see Fig. 6a). Among these features, only a few properties exhibit clear correlations. For example, $\mu _{z,\mathrm{OM}}$ and $\mu _{z,\mathrm{DM}}$ are strongly correlated, as $\mu _{z,\mathrm{DM}}$ contains information from $\mu _{z,\mathrm{OM}}$. These features are also correlated with $\Delta \phi$ because they partially enter Eq. 10. In contrast, the majority of the top 10 features show weak correlations ($|\rho _s| < 0.05$), indicating that SHAP-based feature ranking effectively mitigates multicollinearity among the QM descriptors. This procedure filters out highly correlated and thus noisy features, ultimately leading to improved generalization by leveraging a diverse set of non-redundant descriptors. Moreover, the counterpart $\epsilon _{\mathrm{L,DM}}$ of $\epsilon _{\mathrm{H,DM}}$ does not appear among the top 10 features, whereas $\epsilon _{\mathrm{H,DM}}$ ranks second. This suggests that the surface Fermi level predominantly interacts with $\epsilon _{\mathrm{H,DM}}$, consistent with orbital mixing theory [66]. Consequently, $\epsilon _{\mathrm{H,DM}}$ tends to align with the surface Fermi level, and the resulting Fermi level of the complex system, $E_{\mathrm{f,CPLX}}$, is more strongly associated with $\epsilon _{\mathrm{H,DM}}$ than with $\epsilon _{\mathrm{L,DM}}$, with $\rho _s = 0.8$ and $\rho _s = -0.35$, respectively (see Fig. 6b). To further investigate the synergistic mechanisms of the most important QM features, e.g., $u_{z,\mathrm OM}$ and $\epsilon _\mathrm{H,\mathrm DM}$, in tuning $\Delta \phi$, we analyze their contribution behavior by correlating SHAP values with property distributions (see Fig. 6(c)). In the left panel, the SHAP values of $\mu _\mathrm{z,\mathrm OM}$ exhibit a clear linear correlation with the feature itself: negative $\mu _\mathrm{z,\mathrm OM}$ values yield positive contributions to $\Delta \phi$, and vice versa, with the sign determined by the direction of the surface dipole moment. In general, larger absolute values of $\mu _\mathrm{z,\mathrm OM}$ lead to stronger contributions to $\Delta \phi$, consistent with its $\rho _s$ value. In contrast, the SHAP values of $\epsilon _\mathrm{H,\mathrm DM}$ in the right panel remain nearly constant as $\epsilon _\mathrm{H,\mathrm DM}$ increases from its minimum up to approximately $-5.25\,\mathrm eV$. Beyond this turning point, a linear correlation between SHAP values and $\epsilon _\mathrm{H,\mathrm DM}$ emerges as the feature value increases further.

These behaviors can be understood through the intrinsic interpretability of tree-based models. Owing to the hierarchical splitting process, features used at shallower tree depths acquire greater importance than those applied deeper in the tree, since early splits typically yield larger information gains by partitioning a larger fraction of the dataset. As shown in Fig. 6d, we quantify the frequency with which $\mu _{z,\mathrm OM}$, $\epsilon _\text{H, DM}$ and $\epsilon _\text{H, REC}$ (ranked $4^\mathrm{th}$ in Fig. 5) participate in splits at each tree depth. At the first tree level, the bars corresponding to $\mu _{z,\mathrm OM}$ and $\epsilon _\text{H, DM}$ are markedly higher than that of $\epsilon _\text{H, REC}$, with $\mu _{z,\mathrm OM}$ also significantly exceeding $\epsilon _\text{H, DM}$. At greater depths, $\mu _{z,\mathrm OM}$ and $\epsilon _\text{H, DM}$ continue to participate frequently in splits, albeit with reduced information gain due to the smaller number of remaining data points. Notably, $\epsilon _\mathrm{H,\mathrm DM}$ slightly surpasses $\mu _{z,\mathrm OM}$ in splitting frequency at deeper levels, corresponding to splits that isolate a small number of exceptional outliers. This compensates for the stronger early contribution of $\mu _{z,\mathrm OM}$, resulting in comparable total SHAP contributions for the two features, as reflected in the importance ranking in Fig. 5. Overall, these observations confirm the dominant and widespread importance of $\mu _{z,\mathrm OM}$ and $\epsilon _\text{H, DM}$, with $\mu _{z,\mathrm OM}$ retaining a slightly higher overall ranking. The shorter bar heights of $\epsilon _\mathrm{H,REC}$ are also consistent with its narrowly distributed SHAP values and, consequently, its lower importance. Moreovero, the turning point at $\epsilon _\mathrm{H,\mathrm DM} \approx -5.25\,\mathrm eV$ can be illustrated by the participation of the property values (corresponding to decision borders in tree-based models) at the splitting nodes (see Fig. 6e). The number of borders with values $> -5.25 \, \mathrm eV$ is significantly higher than those $\le -5.25 \, \mathrm eV$, indicating that the values above this threshold appear more frequently in the split decisions. In this regime, the property values are more continuous and lead to a broader range of contribution values, whereas borders $\le -5.25 \, \mathrm eV$ participate much less frequently in the splitting process. Indeed, the cumulative information gain from borders $\le -5.25 \, \mathrm eV$ results in only minor contributions, fluctuating between 0 and $0.1\, \mathrm eV$ to $\Delta \phi$. Contrarily, border $> -5.25 \, \mathrm eV$ yield large contribution with a broad distribution, consistent with the threshold effect shown in the left panel of Fig. 6c. This behavior can be attributed to the energetic alignment between the molecular frontier orbitals and the surface Fermi level in the CPLX system. In particular, $\epsilon _\mathrm{H,\mathrm DM}$ plays a critical role, as evidenced by its stronger correlation with $E_\mathrm{f,CPLX}$ (see Fig. 6b). We therefore hypothesize that when $\epsilon _\mathrm{H,\mathrm DM}$ lies well below the surface Fermi level, the HOMO is energetically inaccessible and induces negligible charge redistribution at the surface, resulting in a minimal impact on work-function modulation. Conversely, when $\epsilon _\mathrm{H,\mathrm DM}$ exceeds the surface Fermi level, substantial charge redistribution can occur, and $\Delta \phi$ is governed by the energetic separation between the HOMO and the surface Fermi level. Finally, this threshold effect may also be influenced by the spatial localization of the frontier orbitals on the molecule [78], which affects their coupling to the surface.

## Conclusions

In the present work, we have introduced MORE-ML, a computational framework that integrates quantum-mechanical (QM) property data of electronic-nose molecular building blocks with machine-learning (ML) methods to predict and interpret the physicochemical mechanisms governing sensing-related properties. This challenging task is addressed by expanding our previously generated MORE-Q dataset into MORE-QX, which spans a significantly larger conformational and property space for interacting systems composed of combinations of body-odor volatilomes (BOVs) and mucin-derived receptors (REC). Based on MORE-QX, we construct a set of binding features (BFs) by computing the adsorption energy ($E_\mathrm{ads}$), work-function change ($\Delta \phi$), and charge transfer ($\Delta Q$). These quantities quantify the impact of BOV–REC interactions on the energy, work function ($\phi$), and charge distribution of the REC–graphene systems. Analysis of the property space spanned by MORE-QX reveals clear evidence of “Freedom of design” in the BF space, i.e., the ability to identify chemically diverse OM–REC–graphene (CPLX) conformations that exhibit a targeted set of BFs. This flexibility arises from the weak correlations observed among most QM properties. Furthermore, property–property correlation analysis highlights the potential of several electronic features to discriminate between similar DM and CPLX conformations, a key requirement for constructing efficient molecular descriptors. Most electronic features included in MORE-QX are invariant with respect to translations, rotations, and atom permutations, thereby satisfying a central requirement for a complete molecular representation suitable for ML-based predictive modeling.

Leveraging these insights within the MORE-ML framework, we define deterministic mappings between the electronic features of molecular building blocks (e.g., OM, REC, and DM systems) and the BFs. These mappings are designed to reduce the computational cost of determining sensing-related properties, as computing QM properties for individual building blocks is significantly less expensive than direct BF calculations. To this end, we performed feature engineering and benchmarked multiple ML regression techniques to identify the optimal set of electronic features for developing accurate and reliable regression models for each BF. In contrast to previous ML studies that primarily emphasize predictive performance, we place strong emphasis on model explainability by combining the intrinsic interpretability of tree-based models with SHapley Additive exPlanations (SHAP) analysis. Indeed, we find that $E_\mathrm{ads}$ is largely governed by the interaction energy between the OM and REC systems, whereas $\Delta Q$ is primarily influenced by charge-related properties, such as dipole and quadrupole moments. In the case of $\Delta \phi$, an interplay emerges between the vertical dipole moment of OM and the HOMO energy of the DM system, reflecting the physical mechanisms underlying the determination of the work function $\phi$. This in-depth investigation reveals the key physicochemical factors governing each BF and thereby establishes a more transparent and navigable pathway through the largely unexplored binding feature space.

From the e-nose sensing materials design perspective, the demonstrated “Freedom of design” in the binding feature space is particularly valuable, as it suggests that sensor sensitivity, baseline stability, and selectivity can be tuned semi-independently through receptor engineering rather than relying on trial-and-error material screening. The finding that adsorption energy, charge transfer, and work function modulation are governed by distinct and weakly correlated electronic descriptors aligns well with practical observations in sensor arrays, where signal amplitude, recovery behavior, and device-to-device variability often decouple. Importantly, the interpretability of the MORE-ML framework provides experimentally actionable guidelines for selecting or synthesizing receptor molecules that target specific transduction mechanisms, thereby reducing empirical optimization cycles. The accuracy and interpretability of our ML models could be further improved by adopting $\Delta$-ML strategies for predicting BFs, as has been successfully demonstrated for other relevant physicochemical properties of complex molecular systems [79–81]. In principle, two possible $\Delta$-ML strategies could be considered for developing a robust baseline model. First, a linear relationship could be established between the most relevant descriptors, identified through SHAP analysis, and the BFs. Second, a mathematical expression involving these descriptors could be constructed using the sure independence screening and sparsifying operator (SISSO) method [82].

Based on the findings presented in this work, we successfully demonstrate a sustainable AI-based framework that reveals multiple sensing mechanisms from a computational perspective [31]. Although MORE-QX is limited to sensing-related properties on graphene surfaces, this comprehensive analysis elucidates the fundamental mechanisms controlling BFs—properties that are strongly linked to sensing performance—through the manipulation of DM properties. However, to extend the applicability and improve the reliability of our regression models to other relevant 2D sensing material substrates, such as two-dimensional MXenes [83, 84] and transition-metal dichalcogenides (TMDs) [85, 86], electronic features describing receptor–substrate interactions must be incorporated into the MORE-ML framework. These augmented descriptors may provide a more appropriate mapping between the property space of e-nose molecular building blocks and the binding-feature space. This, in turn, enables the definition of novel design principles for high-performance, sensitive, and selective molecular receptors, which can subsequently be validated using generative AI approaches or experimental measurements. We note that achieving a full understanding of sensing mechanisms in electronic-nose devices also requires investigating the contact potential between the electrode and the sensing surface (i.e., the Schottky barrier effect [27]), as it may play a dominant role in sensing performance. Therefore, we expect this work to motivate future research aimed at advancing sensing materials by leveraging physical and chemical insights together with deterministic property mappings enabled by the integration of quantum science and interpretable ML regression models.

## Supplementary Information

Supplementary material 1.

## Data Availability

All datasets, source code, running examples, and trained ML models presented in this work are available in the MORE-Q GitHub repository (https://github.com/LiC1117/MORE-Q) and are also publicly available on Zenodo under the DOI https://zenodo.org/records/14720508.
