# Supplementary material for: Towards the design of artificial sensing materials via quantum-informed explainable AI
Source: J Cheminform. 2026 May 29;18:75. doi: 10.1186/s13321-026-01232-3 (PMC13248337; doi:10.1186/s13321-026-01232-3)
Supplement: Supplementary file 1 — Supplementary material 1. [file 13321_2026_1232_MOESM1_ESM.pdf]

Supplementary Information (SI) for:

Towards the Design of Artificial Sensing Materials via Quantum-Informed Explainable AI

Li Chen[1], Leonardo Medrano Sandonas[1,*], Shirong Huang[1], Alexander Croy[2], Gianaurelio Cuniberti[1,3,4,5,*],

[1] *Institute for Materials Science and Max Bergmann Center of Biomaterials, TU Dresden, 01062 Dresden, Germany.*

[2] *Institute of Physical Chemistry, Friedrich Schiller University Jena, 07737 Jena, Germany.*

[3] *Dresden Center for Computational Materials Science (DCMS), TUD Dresden University of Technology, 01062 Dresden, Germany.*

[4] *Cluster of Excellence CARE, TU Dresden and RWTH Aachen, Germany*

[5] *Cluster of Excellence CeTI, TU Dresden, Germany*

[*] Corresponding author: Leonardo Medrano Sandonas (`leonardo.medrano@tu-dresden.de`), Gianaurelio Cuniberti (`gianaurelio.cuniberti@tu-dresden.de`)

1 Property and abbreviation tables

Table S1 List of abbreviations used in the manuscript.

Abbreviation	Definition
BOV	Body odor volatilomes
OM	BOV molecule
REC	Receptor
DM	BOV–receptor dimer
BD	Binding features
OM–REC	BOV–receptor complex
CPLX	BOV–receptor–surface complex
SUB	Receptor–surface substrate system
TR	Training set
TE	Test set
UMAP	Uniform Manifold Approximation and Projection for Dimension Reduction
t-SNE	t-distributed Stochastic Neighbor Embedding
RF	Random Forest
GB	Gradient Boosting Decision Tree
CAT	CatBoost
XGB	XGBoost
LGBM	LightGBM
HOMO	Highest Occupied Molecular Orbital
LUMO	Lowest Unoccupied Molecular Orbital
SHAP	SHapley Additive exPlanations
MORE-Q	Molecular Olfactorial Receptor Engineering by Quantum Mechanics
MORE-QX	Extended Molecular Olfactorial Receptor Engineering by Quantum Mechanics

Table S 2 List of the Quantum-mechanical (QM) properties (and corresponding symbols) taken from MORE-QX dataset analyzed in this work. In the units provided for each of these QM properties, a_0 stands for the atomic unit of length (Bohr radius). Property types are classed according to the building blocks as follow: Monomer (OM, REC), Dimer(DM), complex system (CPLX), and binding feature (BD). A full characterization of QM properties in MORE-QX dataset can be found in the MORE-Q manuscript [1].

Symbol	Property description	Units	Type
$\mu_{\mathrm{z,OM}}$	OM dipole moment z component	Debye	Monomer
$\epsilon_{\mathrm{H,REC}}$	REC HOMO orbital energy	eV	Monomer
$\epsilon_{\mathrm{H,OM}}$	OM HOMO orbital energy	eV	Monomer
μ_{REC}	REC total dipole moment	Debye	Monomer
μ_{OM}	OM scaler total	Debye	Monomer
$Q_{xy,\mathrm{REC}}$	REC quadrupole moment tensor xy component	Buckingham	Monomer
$I_{xy,\mathrm{REC}}$	Inertia moment tensor xy component	amu · Å	Monomer
$\epsilon_{\mathrm{H,DM}}$	OM-REC HOMO orbital energy	eV	Dimer
$\epsilon_{\mathrm{L,DM}}$	OM-REC LUMO orbital energy	eV	Dimer
$\mu_{z,\mathrm{DM}}$	OM-REC dipole moment z component	Debye	Dimer
$\alpha_{\mathrm{s,DM}}$	OM-REC molecular isotropic polarizability	a_0^3	Dimer
μ_{DM}	OM-REC total dipole moment	Debye	Dimer
$\epsilon_{\mathrm{gap,DM}}$	OM-REC HOMO-LUMO gap	eV	Dimer
E_{int}	OM-REC binding energy	eV	Dimer
$E_{f,\mathrm{CPLX}}$	OM-REC-graphene Fermi level	eV	Complex
E_{ads}	Adsorption energy	eV	Binding feature
$\Delta\phi$	Work function change	eV	Binding feature
ΔQ	Bader charge transfer	e	Binding feature

Table S3 Full list of the Quantum-mechanical (QM) properties (and corresponding symbols) used as input electronic features D_{ele}. The units and property type categories provided are the same as those in Tab. S2. Property types are classed according to the building blocks as follow: Monomer (OM, REC), dimer (DM), complex system (CPLX), and binding feature (BD). One property might simultaneously apply to different systems. As a result, 130 features are used as the original features for machine learning models. A full characterization of QM properties in MORE-QX dataset can be found in the MORE-Q manuscript [1].

#	Property	Symbol	Unit	Dimension	System	HDF5 keys
1	Total PBE+D3 energy	E_{tot}	eV	1	OM, REC, DM	'ePBE+D3'
2	Nuclear repulsion energy	E_{nuc}	eV	1	OM, REC, DM	'eNUC'
3	Electronic repulsion energy	E_{ele}	eV	1	OM, REC, DM	'eELE'
4	One electron energy	E_{1e}	eV	1	OM, REC, DM	'e1E'
5	Two electron energy	E_{2e}	eV	1	OM, REC, DM	'e2E'
6	Virial potential energy	E_{pe}	eV	1	OM, REC, DM	'ePE'
7	Virial kinetic energy	E_{ke}	eV	1	OM, REC, DM	'eKE'
8	Exchange energy	E_{x}	eV	1	OM, REC, DM	'eX'
9	Correlation energy	E_{c}	eV	1	OM, REC, DM	'eC'
10	Exchange-correlation energy	E_{xc}	eV	1	OM, REC, DM	'eXC'
11	Total D3 energy	E_{D3}	eV	1	OM, REC, DM	'eD3'
12	Dispersion E6 energy	E_6	eV	1	OM, REC, DM	'eE6'
13	Dispersion E8 energy	E_8	eV	1	OM, REC, DM	'eE8'
14	HOMO energy	ϵ_{H}	eV	1	OM, REC, DM	'eH'
15	LUMO energy	ϵ_{L}	eV	1	OM, REC, DM	'eL'
16	HOMO-LUMO gap	ϵ_{gap}	eV	1	OM, REC, DM	'HLgap'
17	Isotropic molecular C_6 coefficient	C_6	$E_{\text{h}} \cdot a_0^6$	1	OM, REC, DM	'mC6'
18	Total dipole moment	μ	D	3	OM, REC, DM	'vDIP'
19	Scalar total dipole moment	μ_{s}	D	1	OM, REC, DM	'DIP'
20	Rotational spectrum constant	B	MHz	3	OM, REC, DM	'vRS'
21	Rotational dipole moment	μ_{B}	d	3	OM, REC, DM	'vRSDIP'
22	Total quadrupole moment tensor	Q	Buckingham	6	OM, REC, DM	'TQP'
23	Isotropic molecular quadrupole	Q_{s}	Buckingham	1	OM, REC, DM	'mQP'
24	Molecular polarizabillity tensor	α	a_0^3	6	OM, REC, DM	'mTPOL'
25	Molecular isotropic polarizability	α_{s}	a_0^3	1	OM, REC, DM	'mPOL'
26	Radius of gyration	R_{g}	Å	1	OM, REC, DM	'RG'
27	Inertia moment tensor	I_{TS}	amu·Å^2	6	OM, REC, DM	'IM'
28	Atomisation energy	E_{at}	eV	1	OM, REC, DM	'eAT'
29	Binding energy	E_{int}	eV	1	DM	'eBIND'

2 MORE-QX data distribution

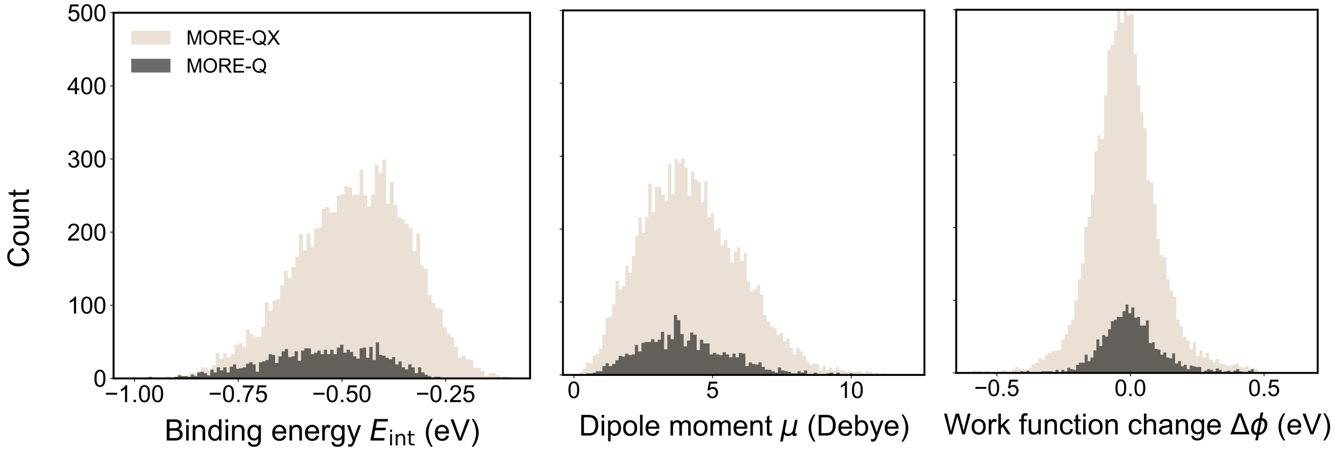

Fig. S1 Three examples for the property distribution comparison between MORE-QX (brown) and MORE-Q (black)

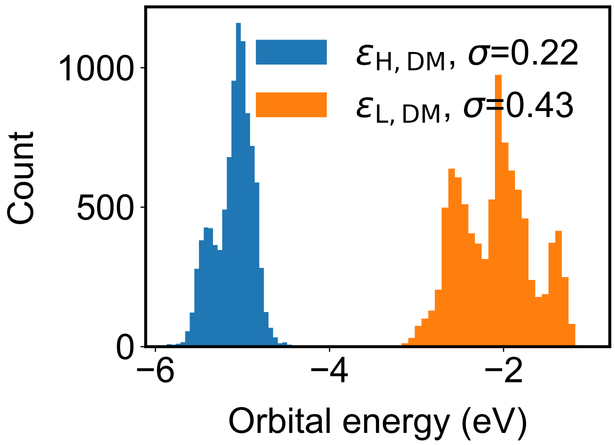

Fig. S2 The distribution of the dimer HOMO ($\epsilon_{H,DM}$, blue) and LUMO ($\epsilon_{L,DM}$, orange) orbital energies. We show the respective variances σ.

3 Tree-based Machine learning models

Tree-based ML models, which belong to the ensemble learning category, are divided into bagging and gradient boosting methods, and their performance towards different regression tasks were benchmarked in Fig. S7. In this section, we introduce the main features for each model regarding regression task. The uniform definition is given as follow:

$$F(x) \;=\; \sum_{m=1}^{M} w_m\, h_m(x;\, \theta_m), \tag{1}$$

where $h_m(x; \theta_m)$ is the mth tree (weak learner) and its structure and leaf values are determined by the hyperparameter θ_m. And the w_m is the weight of the mth. The objective function for training process and the loss function for each tree is defined as:

$$\theta_m = \arg\min_{\theta} \sum_{i=1}^{n} \ell\big(y_i,\; F_{m-1}(x_i) + w_m\, h_m(x_i;\theta)\big) \;+\; \Omega\big(h_m(x;\theta)\big), \tag{2}$$

and output of the model is then updated by:

$$F_m(x) = F_{m-1}(x) + w_m\, h_m(x; \theta_m), \quad F_0(x) = \bar{y}, \tag{3}$$

where $\ell(y, \hat{y})$ is the loss function bewteen ground truth and prediction value and $\Omega(h)$ denotes the complexity of the tree h and regularizes the training process. And the initial residual *i.e.*, output of the 0th results are set to be average of the output $\bar{y}$.

Random forest (RF)

Random forest trains weak learners independently by simply averaging the predictions from all M the individual trees which turns w_m into $\frac{1}{M}$ in Eq. S1. And the model complexity is controlled via hyperparamters such as tree depth, minimum samples per leaf. An explicit regularization term $\Omega(h)$ is typically omitted.

Gradient boosting decision tree (GB)

In GB method, a constant learning rate is allocated to $w_m = \nu \in (0, 1]$. In each training iteration, pseudo-residuals are computed as following:

$$r_{im} = -\frac{\partial \ell(y_i, F_{m-1}(x_i))}{\partial F_{m-1}(x_i)} \tag{4}$$

And new tree $h_m x$ is fitted to the residuals, and then the model is updated via Eq. S3. The regularization term is explicitly given as:

$$\Omega(h) = \gamma T + \frac{1}{2}\lambda \Sigma_j w_j^2, \tag{5}$$

where T denotes the number of leaves, and γ, λ penalize complexity of the tree structre and leaf weights.

XGBoost (XGB)

To enhance training speed and also model stability, the loss function part Eq. S2 is modified by Taylor expansion at the mth prediction for training sample i and hence Eq. S2 turns into:

$$\theta_m = \arg\min_{\theta} \sum_{i=1}^{n} \big(g_i h(x_i;\theta) + \frac{1}{2} h_i h(x_i)^2\big) \;+\; \Omega\big(h_m(x;\theta)\big), \tag{6}$$

where $g_i = \frac{\partial \ell(y_i,\hat{y})}{\partial \hat{y}}\big|_{\hat{y}=F_{m-1}(x_i)}$ and $h_i = \frac{\partial^2 \ell(y_i,\hat{y})}{\partial^2 \hat{y}}\big|_{\hat{y}=F_{m-1}(x_i)}$ are the first and second derivative (gradient and hessian) of the loss ℓ at the mth prediction.

LightGBM (LGBM)& Catboost (CAT)

Built on XGBoost, LGB adapts the histogram and leaf-wise tree growing strategy to improve training speed with fewer memory counts, while CAT employs the oblivious tree by forcing the ical feature on the node splitting within each layer to overcome overfitting problems further.

4 Additional results on "Freedom of design" in the binding feature space

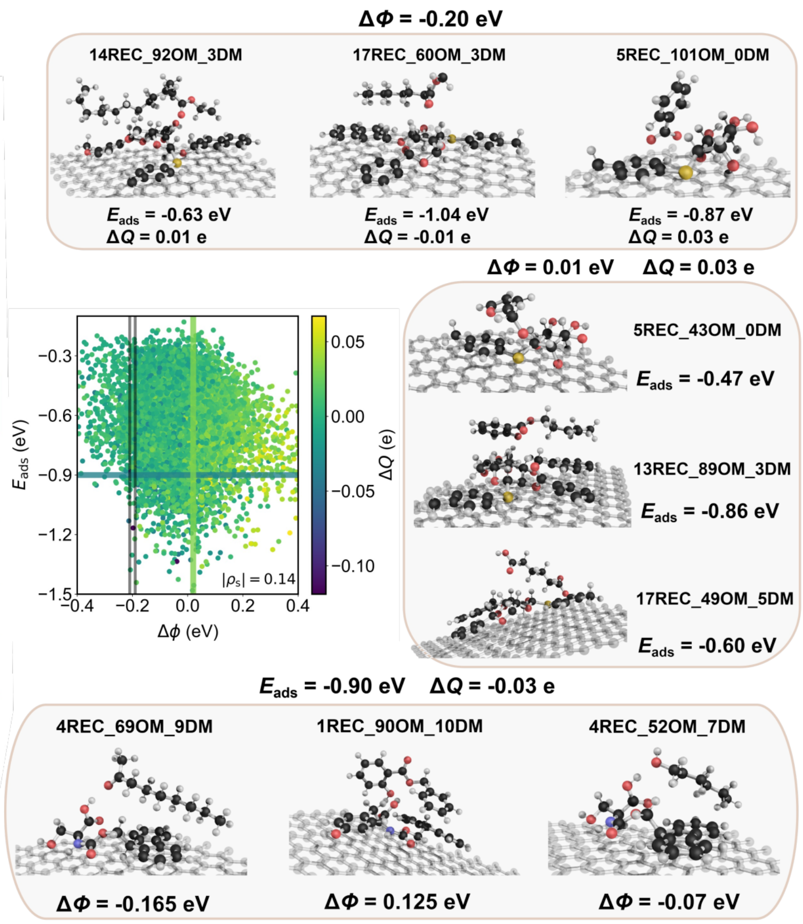

Fig. S3 Examples for design tasks under the freedom of design conjecture. Each system is named by nREC_mOM_lDM, where n, m and l refer to the number of receptor, BOV molecules and their dimer conformer. The full list of BOV and receptor molecules and their number can be viewed in MORE-Q[1]. Top panel: design task for constraining $\Delta\phi = -0.2 \pm 0.01\,\mathrm{eV}$. Right panel: design task for constraining $\Delta\phi = 0.01 \pm 0.01\,\mathrm{eV}$. Bottom panel: constraining the $E_{\mathrm{ads}} = -0.9 \pm 0.01\,\mathrm{eV}$ and $\Delta Q = -0.03 \pm 0.001\,\mathrm{e}$. Middle panel: scatter plot between adsorption energy and work function change, colored by charge transfer.

The scatter plot in the middle panel of Fig. S3 illustrates the correlation between adsorption energy and work function change, yielding a correlation coefficient of $|\rho_{\mathrm{s}}| = 0.14$. This very weak correlation provides an ideal example for exploring the freedom of design conjecture. Therefore, we start firstly with a simple constraint design task given only $\Delta\phi = -0.20 \pm 0.01\,\mathrm{eV}$, in which corresponding the 50th of the negative half distribution of the $\Delta\phi$, as shown in the unfilled dark lines in the scattering plot of Fig. S3. Along the dark lines, the E_{ads} varies in a good range roughly from -0.3 and $-1.1\,\mathrm{eV}$, whose value might be correlating to the DM interacting area especially in weak interaction systems driven by electrostatics or Van der Waals interaction[2]. Contrary to the three systems highlighted in the top panel of Fig. S3, each satisfying identical $\Delta\phi$, display markedly different adsorption energies: the largest OM (ID 92) exhibits $E_{\mathrm{ads}} = -0.63\,\mathrm{eV}$, while the other two denotes -1.04 and $-0.87\,\mathrm{eV}$ with smaller molecular size. The deviation in E_{ads} scaling law might be ascribed to the O-containing pocket formation in DM interaction on the surface. Besides, these DM interaction yields the almost the same $\Delta\phi$ and different ΔQ in both value and sign manifesting the freedom of design conjecture in finding complex structures with low-correlated E_{ads} and ΔQ under one simple $\Delta\phi$

constrain and also reflecting the complexity in correlating the $\Delta\phi$ to the morphological and compositional aspects of the systems. Next, we impose more stringent design constraints by targeting $\Delta\phi = 0.01 \pm 0.01\,\mathrm{eV}$ *i.e.*, $\Delta\phi$ non-dominant cases and relative larger $\Delta Q = 0.03\,\mathrm{eV}$ as shown in the yellow strip in the scattering plot. Under more stringent conditions, as shown in the middle panel, we can still identify systems with tailored E_{ads}. In these cases, the scaling law holds from 5REC–43OM over 13REC–89OM to 17REC–49OM. As a final demonstration, we impose constraints of $E_{\mathrm{ads}} = -0.9 \pm 0.01\mathrm{eV}$ and $\Delta Q = -0.03 \pm 0.001\mathrm{e}$, thereby ensuring identical recovery times and a charge-transfer–dominant mechanism, as indicated by the grey horizontal line in the bottom panel. In these complexes, hydrogen bonding between the OM and REC molecules compensates for variations in interaction-area size, allowing long-chain, pyrene-ring-based, and small-size systems to exhibit identical E_{ads} values. Interestingly, the three systems yield $\Delta\phi$ with both different values and sign under an identical ΔQ, which manifests again the freedom of design again from another perspective.

5 The entire workflow for MORE-ML

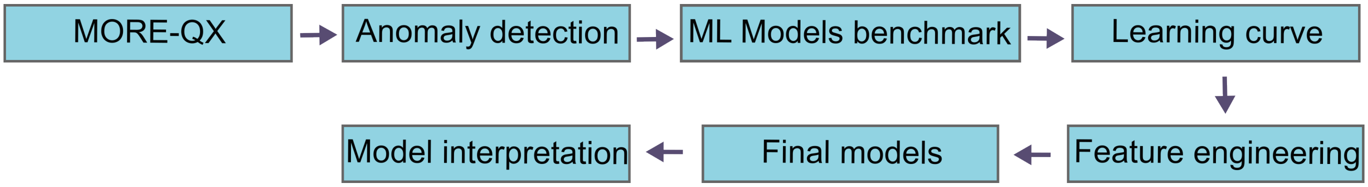

Fig. S4 Overview of the entire Machine learning workflow in MORE-ML.

6 Anomaly detection

During our initial benchmarking runs for predicting adsorption energy (E_{ads}) with XGBoost (XGB), performance remained unsatisfactory after multiple trials, as shown in Fig. S5 (a). Then we checked the geometries of the outliers on Fig. S5 (a). In the example shown in Fig. S5 (b), the OM's dominant interaction is with graphene rather than the receptor. Accordingly, for each system we counted the number of atoms whose distance to the surface is > 3.5Å, which is $\pi - \pi$ stacking distance and defined this quantity as the descriptor $N_{d_{o-s<3.5\text{Å}}}$. The distribution of $N_{d_{o-s<3.5\text{Å}}}$ are depicted in Fig. S5 (c) indicating that there are indeed minor exceptional systems which have unignorable atoms on the OM interacting mainly graphene. The distribution of $N_{d_{o-s<3.5\text{Å}}}$ shown in Fig. S5 (c) reveals a small subset of exceptional systems in which a non-negligible number of OM atoms interact primarily with graphene. To identify the anomalies most responsible for degrading model performance, we add $N_{d_{o-s<3.5\text{Å}}}$ as a 'diagnostic descriptor' into the input feature. As shown in Fig. S5 (d), performance improves substantially relative to Fig. S5 (a), indicating that inclusion of $N_{d_{o-s<3.5\text{Å}}}$ could help tree-based model better classify the data points associated with the new diagnostic descriptor. Therefore, $N_{d_{o-s<3.5\text{Å}}}$ is highly informative and would gain much importance in predicting E_{ads}. SHAP analysis (Fig. S5 (e)) corroborates this, with $N_{d_{o-s<3.5\text{Å}}}$ ranking as the most important feature. Interestingly, although most systems with low $N_{d_{o-s<3.5\text{Å}}}$ contribute only marginally to the model, a subset exerts a disproportionately large influence on the predictions *e.g.*, red points. Next, we examined the clustering of these systems to identify the outliers, on which the $N_{d_{o-s<3.5\text{Å}}}$ is the sole anomalous factor. To this end, we used UMAP for dimensionality reduction because it preserves global structure relevant to cluster formation. Moreover, we embedded SHAP values rather than raw feature values, since SHAP value captures each feature's contribution and provides a more discriminative representation, allowing samples with similar contribution profiles to cluster more clearly. As highlighted in Fig. S5 (g), the major outliers with high $N_{d_{o-s<3.5\text{Å}}}$ cluster in neighboring regions, whereas points with high $N_{d_{o-s3.5<\text{Å}}}$ in Fig. S5 (h) are distributed broadly and do not exhibit a shared similarity structure in the UMAP space based feature value. Therefore, the outliers' SHAP values form a better cluster than the feature values. Therefore, we identified the 932 highlighted data points in Fig. S5 (g) as the anomalies and removed them from our dataset, as they do not contribute to the OM-REC interaction and hence are not significant for the receptor design tasks. In addition, we list the hyperparameter table of XGBoost for reproduction purposes.

Table S4 XGBoost hyperparameter list used for Anomaly detection.

Hyperparameter	Value
`lambda`	0.603225906844846
`alpha`	0.007555374299051771
`colsample_bytree`	0.6000000000000001
`subsample`	0.9
`learning_rate`	0.016
`n_estimators`	2000
`max_depth`	13
`min_child_weight`	62
`random_state`	20240815
`n_jobs`	1

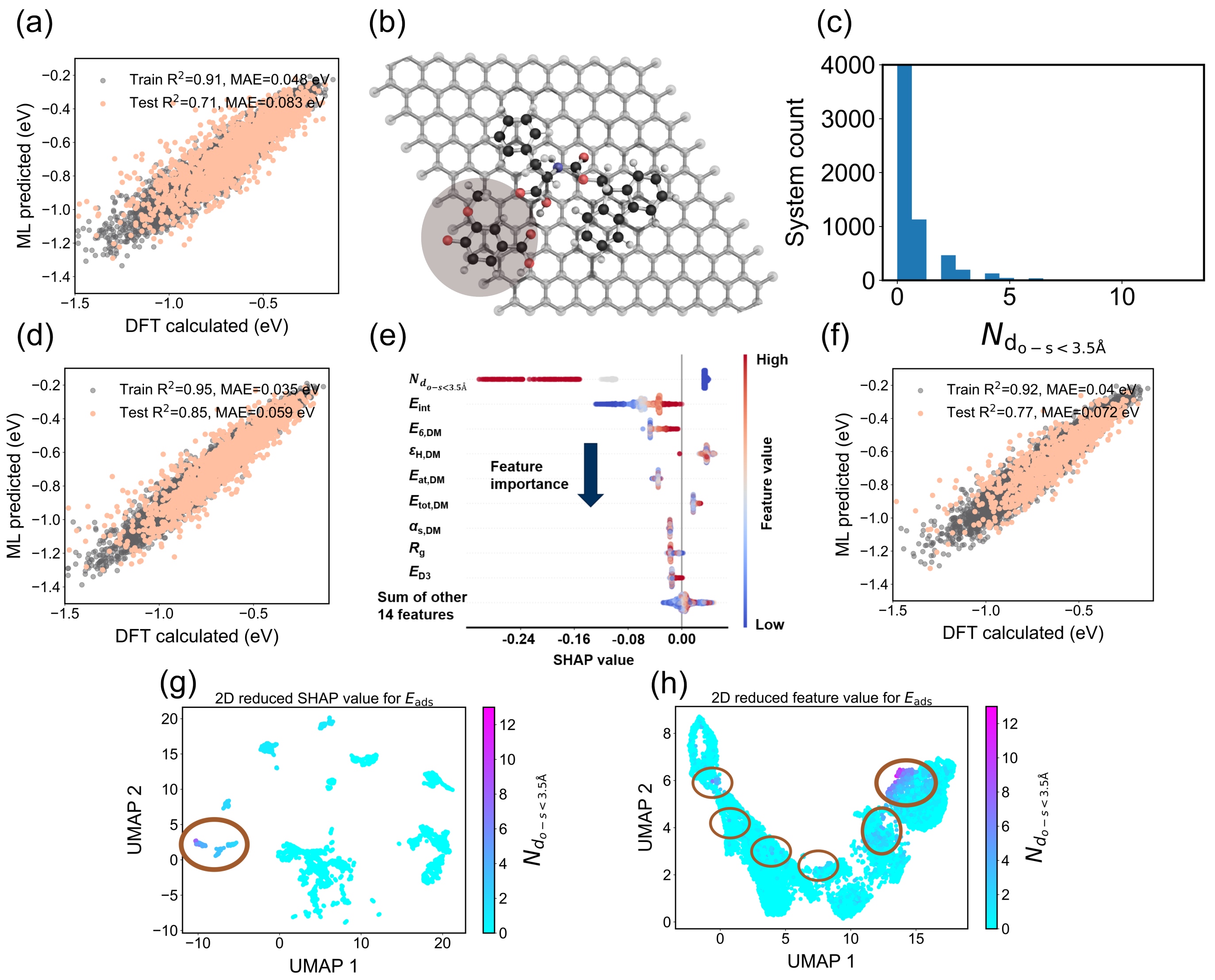

Fig. S5 Anomaly detection workflow for MORE-QX. (a) Adsorption energy E_{ads} prediction using the dimer properties. (b) Atomistic illustration for an anomaly case, where the OM is exposed mainly to graphene. (c) The distribution of the $N_{d_{o-s<3.5\text{Å}}}$ among the 10,411 systems. (d) E_{ads} prediction by adding $N_{d_{o-s<3.5\text{Å}}}$ into the input features under the identical hyperparameters. (e) SHAP analysis beewarms plot from the prediction results in (d). (f) The E_{ads} prediction results after removing the 932 outliers. (g) UMAP plot for clustering the outliers using SHAP value. (h) UMAP plot for clustering the outliers using feature value. The green circles in (g) and (h) are highlighting the location of the outliers.

7 Learning curve

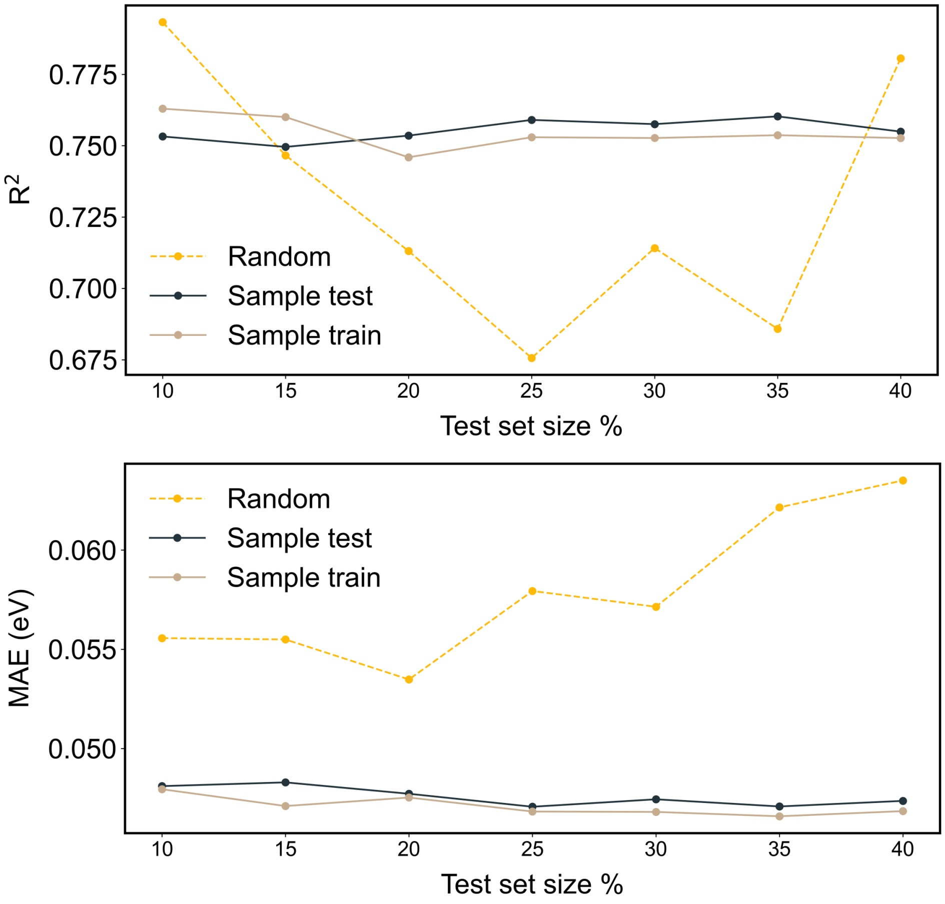

Fig. S6 Learning curve for work function change $\Delta\phi$ prediction with the test set ratio varying from 10% $\sim$ 40% in the whole dataset by Catboost (CAT). Top panel: the R-square score. Bottom panel: the mean absolute error (MAE). The yellow dashed line was generated using random splitting. The black and green dot-line were generated using farthest-point-sampling method by sampling train set (black) and test set (green) on the t-SNE space. For every point, the model's hyperparamters were optimized by Bayesian optimization.

To obtain the best train-test ratio, we conducted a learning curve study, as shown in Fig. S6. Firstly, we can observe the model's performance stability using FPS methods for sampling compared to random splitting (varying from the test data size and no guarantee to the distribution similarity between the train and test set), as the FPS ensures a homogeneous-distribution sampling between the sampled and source dataset. Therefore, we chose the FPS as our splitting method. Secondly, by using FPS, we can choose either the train or test set, and the counterpart is the remaining after sampling. We noticed that this tiny difference would lead to a slight performance discrepancy. As shown in Fig. S6, the performance selecting the test set is generally slightly worse than selecting the train set, as selecting the most representative test set also indicates selecting the most challenging test set. Therefore, concerning our imbalanced dataset and the model performance, we select the splitting ratio 9 : 1 and the train set.

8 Hyperparameter search space

Table S5 Hyperparameter search space of the tree models for Bayesian optimization used in this work.

Model	Hyperparameter	Search Space
XGBoost	lambda, alpha	$[10^{-3}, 10^{-2}, ..., 10.0]$ (Log-uniform)
	colsample_bytree, subsample	$[0.1, 0.2, \ldots, 1.0]$
	learning_rate	$[0.008, 0.010, \ldots, 0.020]$
	n_estimators	$[500, 1000, 3000, 5000, 7000]$
	max_depth	$[2, 4, 6, 8, 10, 12, 14]$
	min_child_weight	$[1, 2, ..., 300]$ (Integer)
RF	n_estimators	$[500, 1000, 3000, 5000, 7000]$
	max_depth	$[2, 4, 6, 8, 10, 12, 14]$
	min_samples_split/leaf	$[2, 4, 6, 8, 10, 12, 14, 16, 18, 20]$
	max_features	$[0.1, 1.0]$ (Float)
GB	n_estimators	$[500, 1000, 3000, 5000, 7000]$
	learning_rate	$[0.008, 0.010, \ldots, 0.020]$
	max_depth	$[2, 4, 6, 8, 10, 12, 14]$
	min_samples_split/leaf	$[2, 4, 6, 8, 10, 12, 14, 16, 18, 20]$
	subsample	$[0.1, 0.2, \ldots, 1.0]$
LightGBM	num_leaves	$[20, 150]$ (Integer)
	max_depth	$[2, 4, 6, 8, 10, 12, 14]$
	learning_rate	$[0.008, 0.010, \ldots, 0.020]$
	n_estimators	$[500, 1000, 3000, 5000, 7000]$
	min_child_samples	$[5, 50]$ (Integer)
	subsample, colsample_bytree	$[0.1, 0.2, \ldots, 1.0]$
CatBoost	iterations	$[500, 1000, 3000, 5000, 7000]$
	learning_rate	$[0.008, 0.010, \ldots, 0.020]$
	depth	$[2, 14]$ (Integer)
	l2_leaf_reg	$[1.0, 10.0]$ (Float)
	border_count	$[32, 255]$ (Integer)

9 Model benchmark information

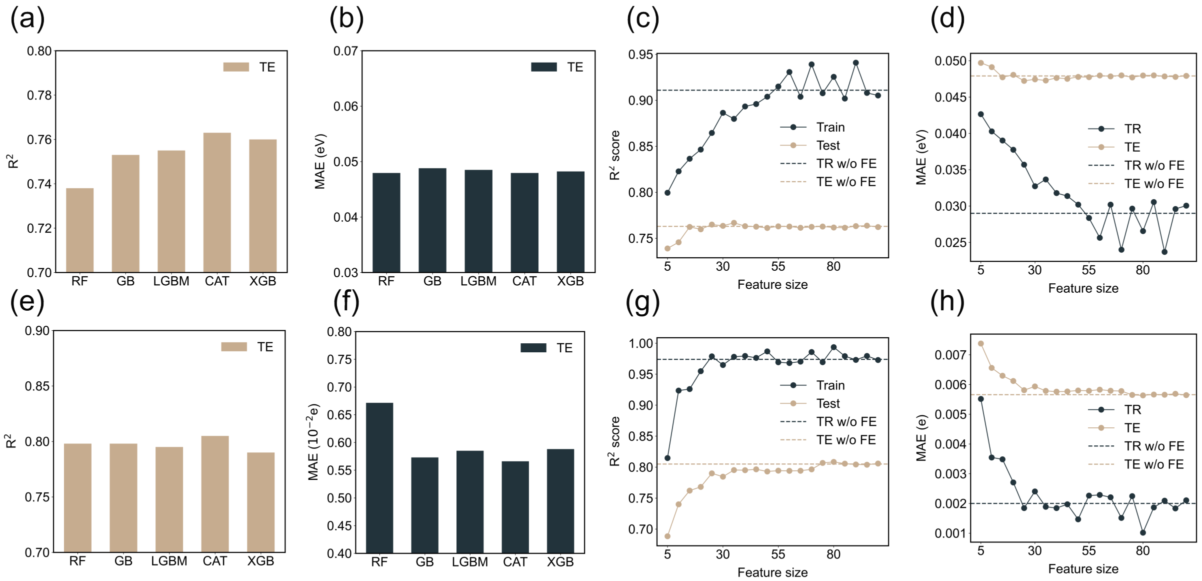

Fig. S7 Model benchmark for work function change $\Delta\phi$ (a)∼(d) and charge transfer ΔQ (e)∼(h). The best model for both binding features remains Catboost (CAT). And the feature size for $\Delta\phi$ and ΔQ accounts for 60 and 80.

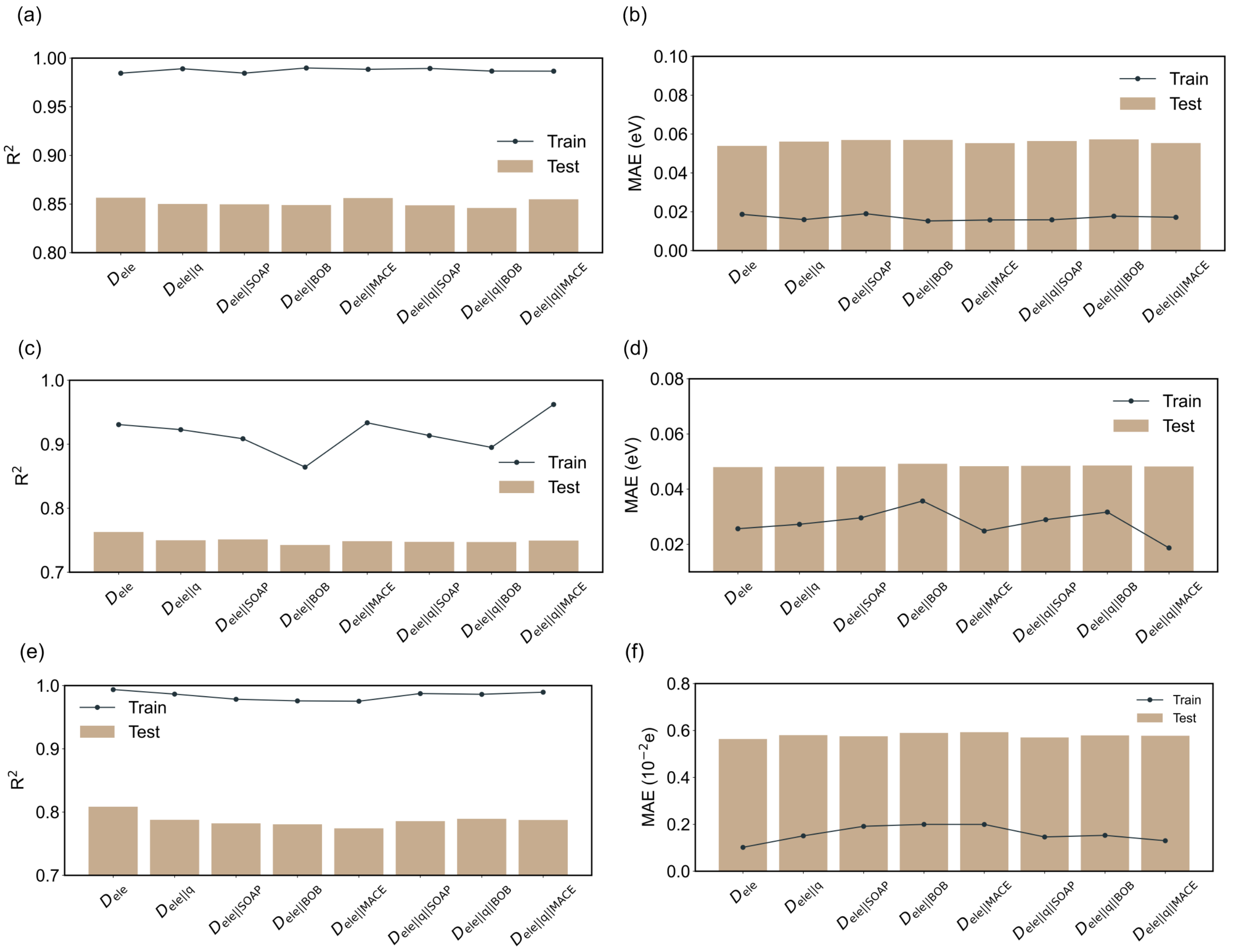

Fig. S8 The model performances by combining the D_{BOB}, D_{SOAP} ,D_{MACE}, D_q and QM properties for (a)∼(b) adsorption energy E_{ads}, (c)∼(d) work function change $\Delta\phi$, and (e)∼(f) charge transfer ΔQ

10 Performance of the geometrical descriptor D_{geo} and Mulliken atomic charge q

As shown in Fig. S8 and S9, we systematically combine the trained models with the geometrical descriptors D_{geo} and Mulliken atomic charges q to evaluate the capability of the vector features. In particular, the principal component analysis is applied to the geometrical descriptors to obtain the most informative expression, and accordingly, they denote D_{BOB}, D_{SOAP} with the length of 63 and 100. And we also involved the MACE descriptor D_{MACE}[3] containing many-body dispersion interaction information. In addition, the atomic Mulliken charge D_q has been handcrafted similarly to BOB descriptors. And the length of Mulliken atomic charge q and MACE descriptor account for 87 and 256, taking up of the total data number with a reasonable ratio of $\sim 4\%$. We added these input features and retrained the models again. The results for all binding feature predictions with D_{geo} and q are depicted in Fig. S8 and S9.

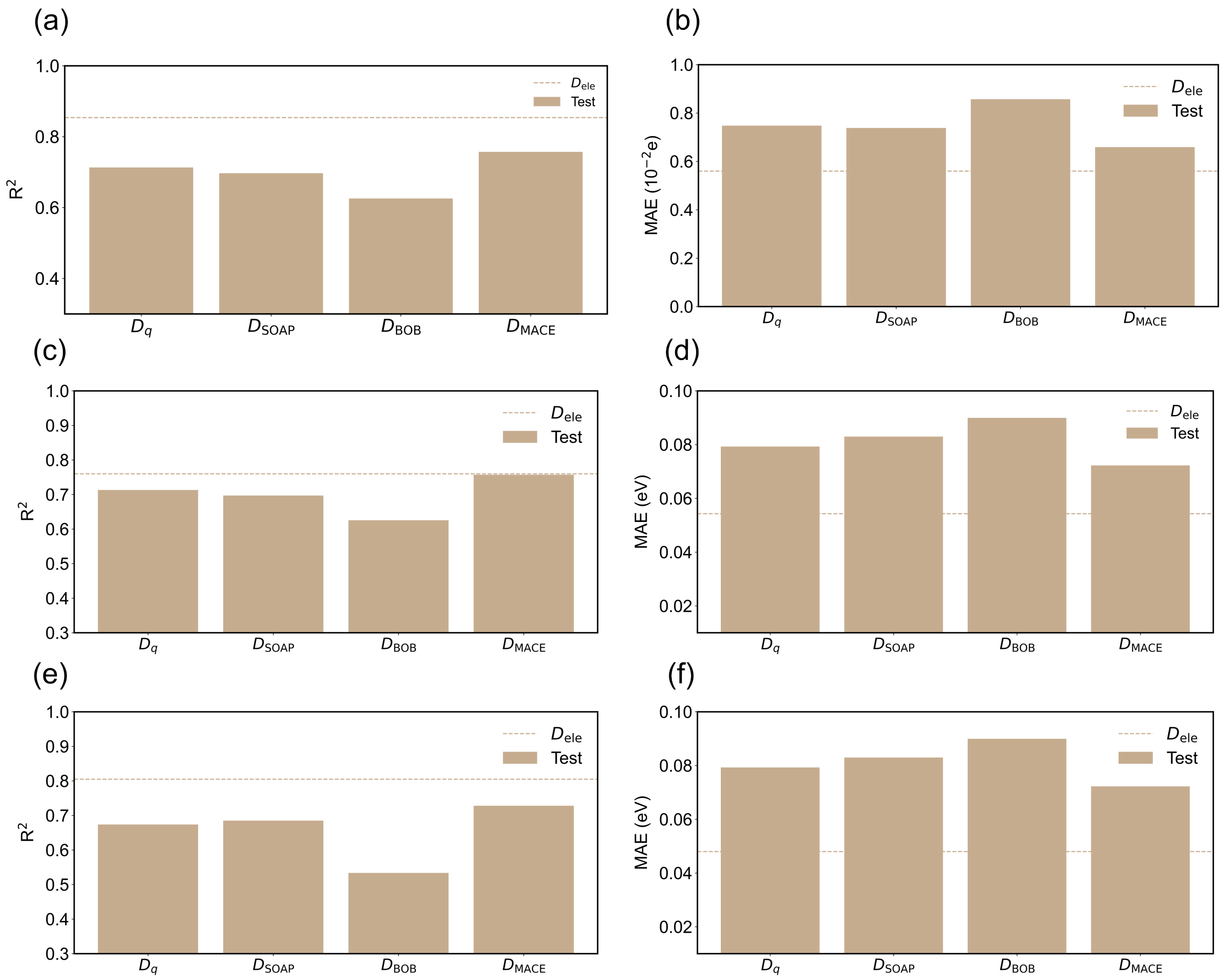

Fig. S9 The same as Fig. S8 but only using these features without electronic properties.

11 Metrics for final models

Table S6 Final Catboost models metrics for binding feature prediction.

	E_{ads}	$\Delta\phi$	ΔQ
**Hyperparameters**			
iterations	7000	5000	7000
learning_rate	0.02	0.01	0.02
depth	7	7	7
l2_leaf_reg	9.240	2.387	1.341
border_count	178	147	169
**Train set**			
R^2	0.984	0.931	0.994
MAE	0.019	0.026	0.001
RMSE	0.024	0.033	0.001
**Test set**			
R^2	0.857	0.763	0.808
MAE	0.054	0.048	0.006
RMSE	0.078	0.064	0.008

12 Explanation of work function change $\Delta\phi$ prediction

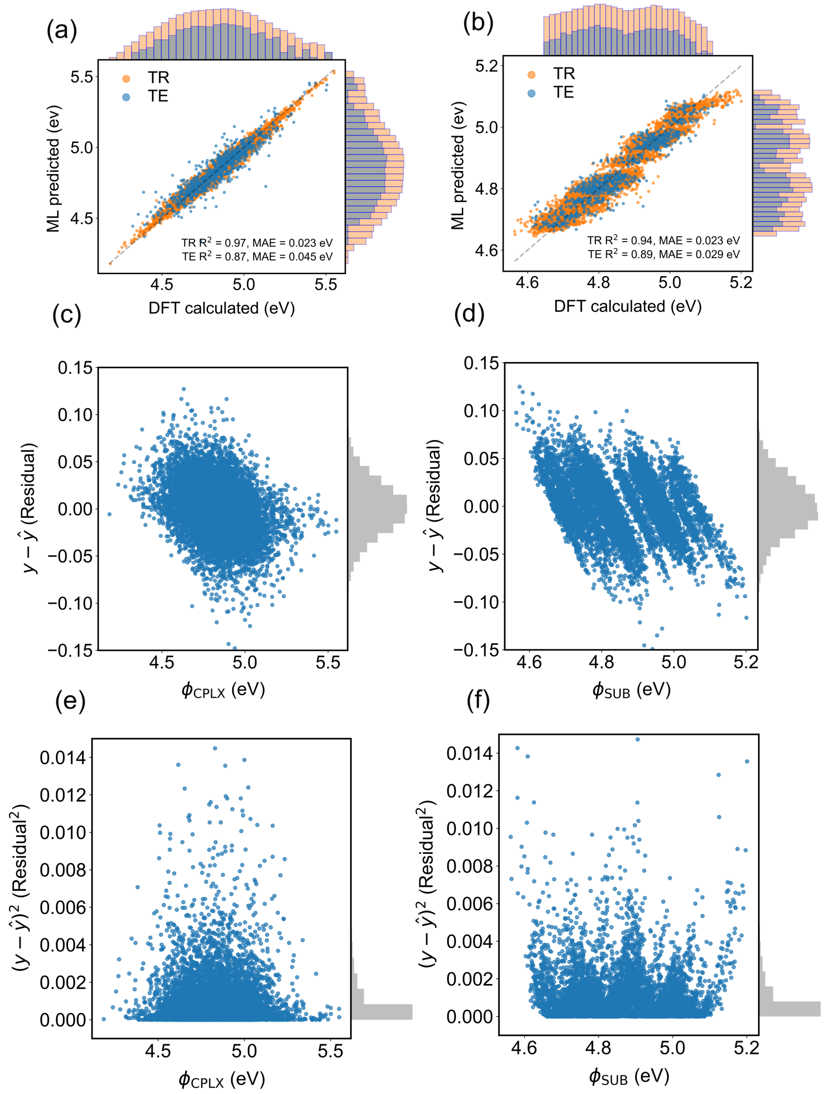

Fig. S10 (a)∼(b) parity plot for ϕ_{CPLX} and ϕ_{SUB} predictions. (c)∼(d) scattering plot for residual vs. ϕ_{CPLX} and ϕ_{SUB}. (e)∼(f) scattering plot for residual's square vs. ϕ_{CPLX} and ϕ_{SUB}.

As shown in Fig. S10 (a) and (b), the individual predictions for ϕ_{CPLX} and ϕ_{SUB} present good performance, while the parity plot of ϕ_{SUB} shows an unusual pattern. To figure out for ϕ_{SUB}'s abnormal prediction behavior, we plotted the residual between the work function and the ML-predicted work function value. In Fig. S10 (c)∼(d), residual vs ϕ_{SUB} exhibit a surprisingly bunch of rod-like shapes with linear correlations, while ϕ_{CPLX} does not show any specific pattern.

The oscillating behavior varying from $-0.1 \sim 0.1$eV offsets to a total tiny error when taking the residual average. Therefore, the residual square also exhibits an abnormal pattern, as shown in Fig. S10 (f). This pattern of residual of ϕ_{SUB} might be owing to the lack of diversity of the receptor-surface leading to this systematic error, and hence imperfect prediction of the work function change $\Delta\phi$. Future work might be focused on expanding the receptor's diversity.

13 Comparing PBE and PBE0 for the calculation of binding features

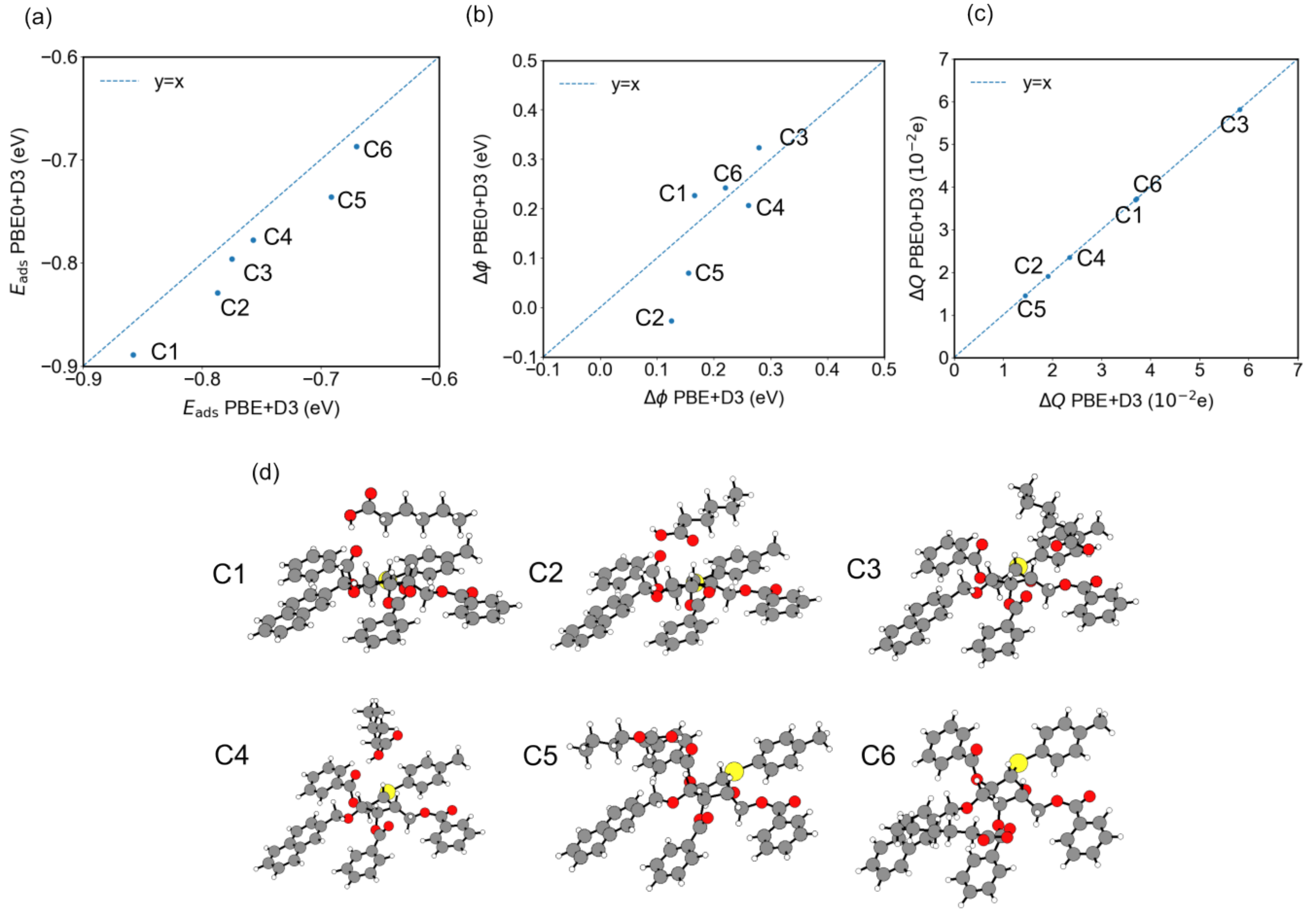

Fig. S11 Comparison of binding features for the 18REC–50OM combination computed at two different levels of theory, PBE+D3 and PBE0+D3: (a) adsorption energy (E_{ads}), (b) work function change ($\Delta\phi$), and (c) charge transfer (ΔQ). (d) Ball-and-stick representations of the six conformers (C1–C6) with the lowest interaction energies for the 18REC–50OM combination.

14 Structural change dependence of electronic properties

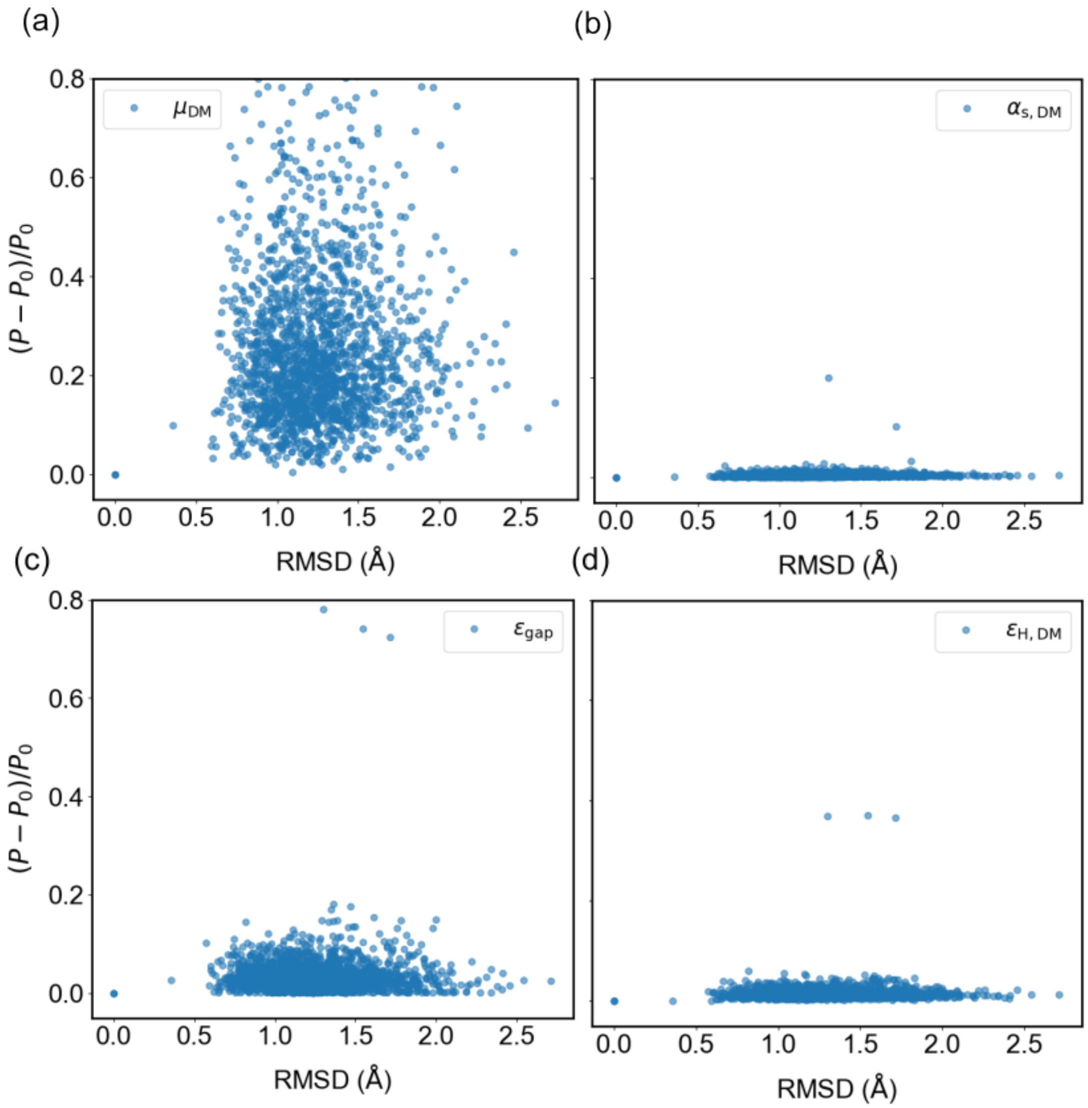

Fig. S12 Variation of electronic properties (P) as a function of the root-mean-square deviation (RMSD) for all dimer configurations (BOV-receptor) contained in the MORE-QX dataset. Results are shown for $P =$ (a) μ_{DM}, (b) $\alpha_{\mathrm{s,DM}}$, (c) ϵ_{gap}, and (d) $\epsilon_{\mathrm{H,DM}}$. Both the property change ratio and the RMSD were calculated with respect to the lowest-energy diemr conformation, with P_0 denoting the reference property value.

References

[1] Li Chen, Leonardo Medrano Sandonas, Philipp Traber, Arezoo Dianat, Nina Tverdokhleb, Mattan Hurevich, Shlomo Yitzchaik, Rafael Gutierrez, Alexander Croy, and Gianaurelio Cuniberti. MORE-Q, a dataset for molecular olfactorial receptor engineering by quantum mechanics. *Sci. Data*, 12(1):324, 2025.

[2] Hans Jürgen Kreuzer and Zbigniew W Gortel. *Physisorption kinetics*, volume 1. Springer Science & Business Media, 2012.

[3] Dávid Péter Kovács, J Harry Moore, Nicholas J Browning, Ilyes Batatia, Joshua T Horton, Yixuan Pu, Venkat Kapil, William C Witt, Ioan-Bogdan Magdau, Daniel J Cole, et al. Mace-off: Short-range transferable machine learning force fields for organic molecules. *J. Am. Chem. Soc.*, 147(21):17598–17611, 2025.
